# PRMT5-Dependent Methylation of the TIP60 Coactivator RUVBL1 Is a Key Regulator of Homologous Recombination

**DOI:** 10.1016/j.molcel.2017.01.019

**Published:** 2017-03-02

**Authors:** Thomas L. Clarke, Maria Pilar Sanchez-Bailon, Kelly Chiang, John J. Reynolds, Joaquin Herrero-Ruiz, Tiago M. Bandeiras, Pedro M. Matias, Sarah L. Maslen, J. Mark Skehel, Grant S. Stewart, Clare C. Davies

**Affiliations:** 1 Institute of Cancer and Genomic Sciences, College of Medical and Dental Sciences, University of Birmingham, Birmingham B15 2TT, UK; 2 Instituto de Biologia Experimental e Tecnológica, 2780-157 Oeiras, Portugal; 3 Instituto de Tecnologia Química e Biológica António Xavier, Universidade Nova de Lisboa, 2780-157 Oeiras, Portugal; 4 MRC Laboratory of Molecular Biology, Francis Crick Avenue, Cambridge Biomedical Campus, Cambridge CB2 0QH, UK

**Keywords:** PRMT5, RUVBL1, arginine methylation, homologous recombination, DNA repair, TIP60, 52BP1

## Abstract

Protein post-translation modification plays an important role in regulating DNA repair; however, the role of arginine methylation in this process is poorly understood. Here we identify the arginine methyltransferase PRMT5 as a key regulator of homologous recombination (HR)-mediated double-strand break (DSB) repair, which is mediated through its ability to methylate RUVBL1, a cofactor of the TIP60 complex. We show that PRMT5 targets RUVBL1 for methylation at position R205, which facilitates TIP60-dependent mobilization of 53BP1 from DNA breaks, promoting HR. Mechanistically, we demonstrate that PRMT5-directed methylation of RUVBL1 is critically required for the acetyltransferase activity of TIP60, promoting histone H4K16 acetylation, which facilities 53BP1 displacement from DSBs. Interestingly, RUVBL1 methylation did not affect the ability of TIP60 to facilitate ATM activation. Taken together, our findings reveal the importance of PRMT5-mediated arginine methylation during DSB repair pathway choice through its ability to regulate acetylation-dependent control of 53BP1 localization.

## Highlights

• PRMT5 is a regulator of homologous recombination-mediated double-strand break repair
• PRMT5 methylates RUVBL1 at R205, regulating TIP60-mediated histone acetylation
• Loss of RUVBL1 methylation leads to 53BP1 retention at break ends
• Arginine methylation crosstalks with histone acetylation to regulate repair pathway choice

## Graphical Abstract

**Figure:**
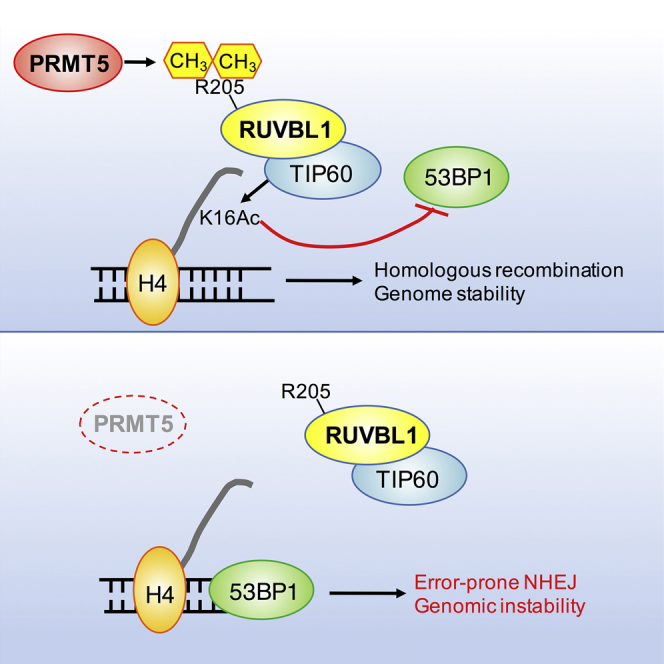

## Teaser

Clarke et al. show that methylation of RUVBL1 by the arginine methyltransferase PRMT5 is required for homologous recombination-mediated double-strand break repair by promoting TIP60-mediated histone H4K16 acetylation. Loss of PRMT5 activity and defective RUVBL1 methylation leads to 53BP1 retention, increased sensitivity to DNA damaging agents, and genome instability.

## Introduction

The genome is constantly being challenged with exogenous and endogenous stresses that induce DNA lesions that must be repaired to maintain genomic stability. An inability to do so leads to mutagenic events that predispose individuals to numerous pathological conditions, including cancer and neurological dysfunction. Cells have therefore developed a complex array of systems that can rapidly sense, respond to, and repair a large number of distinct DNA lesions. Of these, the most deleterious form of DNA damage is the double-strand break (DSB), which, if left unresolved, can result in a loss of genetic material. DSB repair is achieved through two main pathways: homologous recombination (HR) and non-homologous end joining (NHEJ). NHEJ can occur throughout the cell cycle and can be error prone, while HR is error free but requires a sister chromatid as a template and, therefore, only occurs during late S/G2. The main determinant for HR-mediated repair is resection of the DNA DSB end, which is controlled by a 53BP1-containing protein complex containing Rif1, PTIP, and Rev7 (Panier and Boulton, 2014). While 53BP1 has been implicated in potentiating NHEJ-dependent repair of distally located DSBs, its primary function is to protect DSB ends from over-processing by the DNA end resection machinery (Anderson et al., 2001, Mallette et al., 2012, Schultz et al., 2000). If the damage is too complex and a sister chromatid is present, CDK2-mediated phosphorylation of CtIP facilitates the recruitment of BRCA1, displacing 53BP1, enabling end processing and the generation of long stretches of single-stranded DNA (ssDNA). These structures are first coated by replication protein A (RPA), which is later displaced by the RAD51 protein, the main facilitator of strand invasion and recombination (Panier and Boulton, 2014).

While the complexity and sheer number of proteins involved in NHEJ- and HR-mediated DSB repair has long been appreciated, there is a growing understanding that higher-order chromatin structure significantly impacts DSB repair. In this respect, chromatin can have a dual role, sometimes acting as a physical barrier impeding repair or, conversely, offering a platform for DNA repair protein complex recruitment. Thus, chromatin remodeling and post-translational modification of histones and chromatin-bound proteins are now being recognized as important mechanisms integrating local chromatin architecture with repair pathway choice. This is perhaps most clearly illustrated by the recruitment of 53BP1. 53BP1 possesses a Tudor domain in tandem with a UDR domain that is specifically able to read H4K20me2 marks in combination with ubiquitylated histone H2A (Fradet-Turcotte et al., 2013). However, H4K20me2 is a highly abundant, stable histone mark (Pesavento et al., 2008); therefore, to prevent inappropriate recruitment of 53BP1 in the absence of DSBs, H4K20me2 is shielded by a second Tudor domain-containing protein, JMJD2A. DSB formation induces activation of the ATM-signaling pathway, leading to the recruitment of the E3 ligases RNF8 and RNF168, which polyubiquitylate both H2A and JMJD2A, the latter of which triggers its degradation (Mallette et al., 2012). Subsequently, 53BP1 is rapidly recruited within minutes to DSB ends via the exposed H4K20me2 and ubiquitylated H2A marks in an RNF8- and RNF168-dependent manner (Doil et al., 2009, Mailand et al., 2007, Panier and Boulton, 2014, Stewart et al., 2009). Chromatin modifications also play a central role in HR-mediated ejection of 53BP1. A key player regulating this process is the TIP60 DNA repair complex, which has core chromatin remodeling and acetyltransferase activity (Ikura et al., 2000, Sun et al., 2005) and appears to have multiple roles in regulating 53BP1 ejection. Histone H3K9me3 recruits TIP60, promoting the acetylation of histone H4K16, which disrupts 53BP1 binding by affecting salt bridge formation between unmodified H4K16 residues and the 53BP1 Tudor domain (Sun et al., 2009, Tang et al., 2013). In addition to this, it has been shown that a combination of TIP60-mediated H2AK15 acetylation and recruitment of the TIP60 complex component MBTD1 to H4K20me2 facilitates 53BP1 mobilization from break ends (Jacquet et al., 2016). It is therefore becoming increasingly clear that protein modifications act as dynamic platforms promoting the assembly and disassembly of protein complexes, modulating enzymatic activity, and signaling pathway choice.

One modification that we know very little about, particularly within the context of the DNA damage response, is arginine methylation. Protein arginine methyltransferases (PRMTs) catalyze mono- and dimethylation of the guanidino group of the arginine residue using S-adenosyl methionine (SAM) as a methyl donor. Dimethylation can occur asymmetrically (ADMA), with two methyl groups placed onto one of the terminal nitrogen atoms of the guanidino group, or symmetrically (SDMA), where one methyl group is placed onto each of the terminal nitrogen atoms. Recently, a number of studies have demonstrated a role for the main asymmetric arginine methyltransferase PRMT1 in the maintenance of genomic stability and the DNA damage response (Auclair and Richard, 2013). In contrast, only three proteins associated with DNA repair and replication have been identified as PRMT5 substrates (Auclair and Richard, 2013), and a direct role for PRMT5 in regulating DNA repair has not been determined. To address this, we took a systemic approach to analyze the contribution of PRMT5 to DSB repair, and we identified the AAA+ ATPase RUVBL1 (Pontin/Tip49) as a PRMT5 interactor. RUVBL1 and its binding partner RUVBL2 are present in a number of separate high molecular weight nuclear complexes, containing TIP60, SRCAP, or INO80, that regulate a variety of cellular processes, including the DSB response (Alatwi and Downs, 2015, Dong et al., 2014, Gospodinov et al., 2011, Ikura et al., 2000, Jha et al., 2008, Murr et al., 2006, Sun et al., 2005, Tang et al., 2013, Wu et al., 2007). Significantly, we found that PRMT5-dependent methylation of RUVBL1 was required for DSB repair by promoting TIP60-mediated acetylation of H4K16 and 53BP1 removal from sites of damage. Our results thus demonstrate the importance of arginine methylation for DSB pathway choice, and we identify a mechanism by which arginine methylation can specifically direct the activity of a critical DNA repair complex that is known to have multiple roles within the DNA damage response.

## Results

### PRMT5 Regulates Homologous Recombination-Mediated DSB Repair

**Figure 1:**
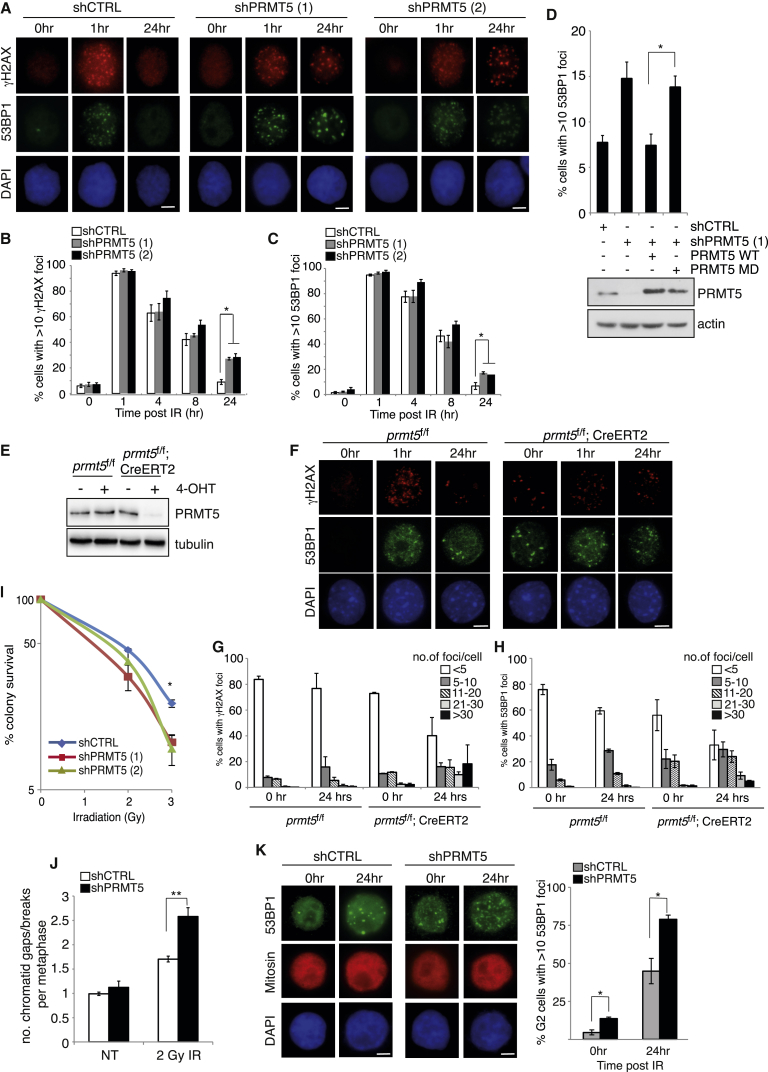
PRMT5 Is Required for Effective Double-Strand Break Repair (A) HeLa-shCTRL, shPRMT5 hairpin 1 (1), and shPRMT5 hairpin 2 (2) cells were stimulated with 3Gy IR and co-stained for γH2AX and 53BP1 foci at the time points indicated. (B and C) Quantification of (A) is shown (mean ± SEM; n = 3). (D) Reconstitution of PRMT5-depleted HeLa cells with wild-type or catalytically inactive (MD) PRMT5. Quantification of 53BP1 foci 24 hr after IR (3 Gy) is shown (mean ± SEM; n = 3). (E) Primary, non-immortalized *prmt5*^f/f^ and *prmt5*^f/f^;CreERT2 mouse embryonic fibroblasts (MEFs) were cultured in the absence or presence of 4-hydroxy-tamoxifen (4-OHT; 500 nM for 24 hr), followed by 4 days in 4-OHT-deficient media, and then immunoblotted for PRMT5. (F–H) Cells were stimulated with 5Gy IR and co-stained for γH2AX and 53BP1 foci at the time points indicated (F). Quantification of γH2AX (G) and 53BP1 (H) foci is shown (mean ± SEM). (I) HeLa-shPRMT5 cells were exposed to increasing doses of IR and cell viability was determined by colony survival assay (mean ± SD; n = 3). (J) Analysis of chromatid gaps and breaks per metaphase is shown (mean ± SEM; ^∗∗^p < 0.0098; n = 3). (K) Quantification of 53BP1 foci in mitosin-positive (late S/G2) cells 24 hr after 3Gy IR is shown (mean ± SEM; n = 3). Scale bar, 10 μm. See also Figure S1.

Recent reports have indicated a role for PRMT5-dependent arginine methylation in Okazaki fragment maturation and replication stress (Guo et al., 2010, He et al., 2011). However, very little is known about whether PRMT5 plays a role in regulating DSB repair. To address this, we generated two PRMT5-knockdown HeLa cell lines each expressing distinct small hairpin shRNA sequences targeting PRMT5 (shPRMT5 [1] and shPRMT5 [2]; Figure S1A), exposed them to ionizing radiation (IR), and then monitored DSB repair by γH2AX/53BP1 foci resolution, two well-established markers of DNA DSBs. As expected, within 24 hr, control cells were able to effectively repair IR-induced DNA lesions, as indicated by the clearance of γH2AX and 53BP1 foci. In contrast, silencing of PRMT5 resulted in a persistence of γH2AX and 53BP1 foci at early (4 hr) and late (24 hr) time points, suggesting a role for PRMT5 in regulating DNA repair processes (Figures 1A–1C and S1B). These effects were also observed in cell lines transiently depleted for PRMT5 (Figure S1C), indicating that this phenotype is unlikely to be attributable to shRNA off-target effects. Importantly, this defect in DNA damage foci clearance could be restored by re-expressing wild-type PRMT5, but not catalytically inactive PRMT5 (PRMT5-G367A/R368A) (Pal et al., 2004), implying that the catalytic activity of PRMT5 is required for effective DSB repair (Figures 1D and S1D). Consistent with shRNA-mediated depletion of PRMT5, genetic deletion of PRMT5 also resulted in delayed clearance of γH2AX and 53BP1 foci after exposure to IR (Figures 1E–1H, S1E, and S1F). Importantly, although PRMT5-null mouse embryonic fibroblasts (MEFs) exhibited spontaneous DNA damage, as evident from γH2AX and 53BP1 foci formation in undamaged cells (Figures 1F–1H), the number of unrepaired foci per cell was far higher in PRMT5-null MEFs 24 hr after IR (Figures 1G and 1H), again indicating a defect in DSB repair. In keeping with this, PRMT5-depleted cells exhibited an increased sensitivity to IR (Figures 1I and S1H) and a failure to properly repair IR-induced chromatid breaks (Figures 1J and S1G).

The proportion of PRMT5-depleted cells retaining unrepaired DSBs at 24 hr post-irradiation was more reminiscent of a HR defect than aberrant NHEJ-mediated DSB repair (Beucher et al., 2009, Shibata et al., 2011). To examine this more closely, we assessed the resolution of IR-induced DSBs only in G2 cells, using aphidicolin to block the transit of any damaged S-phase cells into G2. Strikingly, PRMT5 knockdown led to a significant defect in the ability to repair G2 DSBs, as judged by the presence of 53BP1 foci at 24 hr following the exposure of cells to a low dose of IR (Figure 1K). This observation is consistent with PRMT5 playing a role in HR-dependent DSB repair.

**Figure 2:**
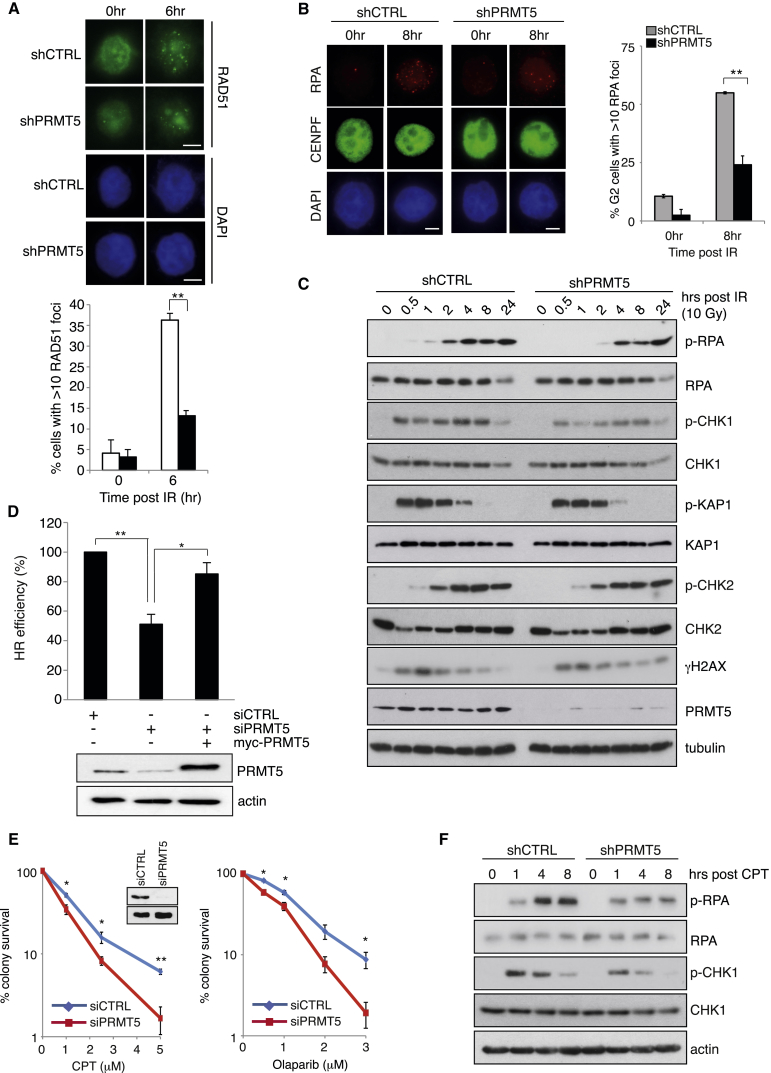
PRMT5 Promotes Homologous Recombination-Mediated DSB Repair Independently of ATM Activation (A) HeLa-shCTRL and shPRMT5 cells were stimulated with 3Gy IR and RAD51 foci analyzed 6 hr later. Quantification of number of cells with >10 RAD51 foci is shown below (mean ± SEM; ^∗∗^p = 0.006; n = 3). (B) Quantification of RPA foci in CENPF-positive (late S/G2) cells 8 hr after 3Gy IR is shown (mean ± SEM; ^∗∗^p = 0.006; n = 3). (C) Time course of phosphorylated and total protein levels is shown. (D) U2OS-DR3 cells were treated with siRNA as indicated before transfection of *I*-*Sce1* and myc-PRMT5. The number of GFP-positive cells (HR proficient) was determined by flow cytometry (mean ± SD; n = 3). (E) PRMT5-depleted cells were treated with increasing doses of camptothecin (CPT; left panel) or Olaparib (right panel), and survival was determined by colony assay (mean ± SD; n = 3). (F) Cells were treated with CPT (1 μM) for the time points indicated and immunoblotted for phosphorylated and total proteins. Scale bar, 10 μm. See also Figure S2.

A key step in the decision to proceed with HR-mediated DSB repair is the 5′-3′ resection of DNA ends. Eviction of 53BP1 enables DNA resection and the production of long stretches of ssDNA that are first coated by RPA, which is later displaced by RAD51 (Panier and Boulton, 2014). To investigate the proposed role for PRMT5 in promoting HR, we determined what impact the loss of PRMT5 had on the ability of cells to generate ssDNA at sites of DSBs and to recruit RAD51 and BRCA1. Notably, PRMT5 depletion significantly reduced RAD51 and BRCA1 recruitment to sites of DNA damage (Figures 2A, S2A, and S2B), without affecting RAD51 expression levels and cell-cycle distribution (Figures S2C and S2D). To ascertain whether the failure of PRMT5-depleted cells to efficiently form IR-induced RAD51 foci resulted from reduced DSB resection or an inability to load RAD51 onto ssDNA generated during resection, we examined the capability of these cells to form RPA foci, as a marker of resection-dependent ssDNA. In support of the former hypothesis, depletion of PRMT5 greatly reduced the number of cells displaying RPA foci, with a concurrent defect in ATR-signaling events, such as RPA S4/S8 and CHK1 phosphorylation (Figures 2B and 2C), suggesting that arginine methylation is regulating DNA DSB end resection. In contrast, activation of the ATM-signaling cascade, determined by the phosphorylation of ATM and its downstream substrates CHK2 and KAP1, was unaffected by PRMT5 depletion (Figures 2C and S2E). In keeping with this, PRMT5 depletion retained the ability to activate the ATM-dependent G2/M checkpoint (Figure S2F). Thus, ATM activation appears to be independent of PRMT5 after IR. Finally, by using the I-SceI GFP HR reporter assay (Gunn and Stark, 2012), we observed a significant decrease in HR-dependent repair in cells lacking PRMT5 expression that could be restored with ectopic expression of small interfering RNA (siRNA)-resistant myc-PRMT5 (Figure 2D).

To further substantiate that PRMT5 is an important component of HR-mediated repair, we treated cells with camptothecin (CPT) and Olaparib, two genotoxic agents that induce DNA lesions specifically repaired by HR (Arnaudeau et al., 2001, McCabe et al., 2006). Analogous to IR-induced damage, knockdown of PRMT5 sensitized cells to both CPT and Olaparib (Figure 2E) and reduced CPT-induced RPA and CHK1 phosphorylation (Figure 2F). Together, these data indicate that PRMT5 functions to promote DNA DSB end resection and HR-dependent DSB repair.

### The AAA+ ATPase RUVBL1 Is a Substrate of PRMT5

**Figure 3:**
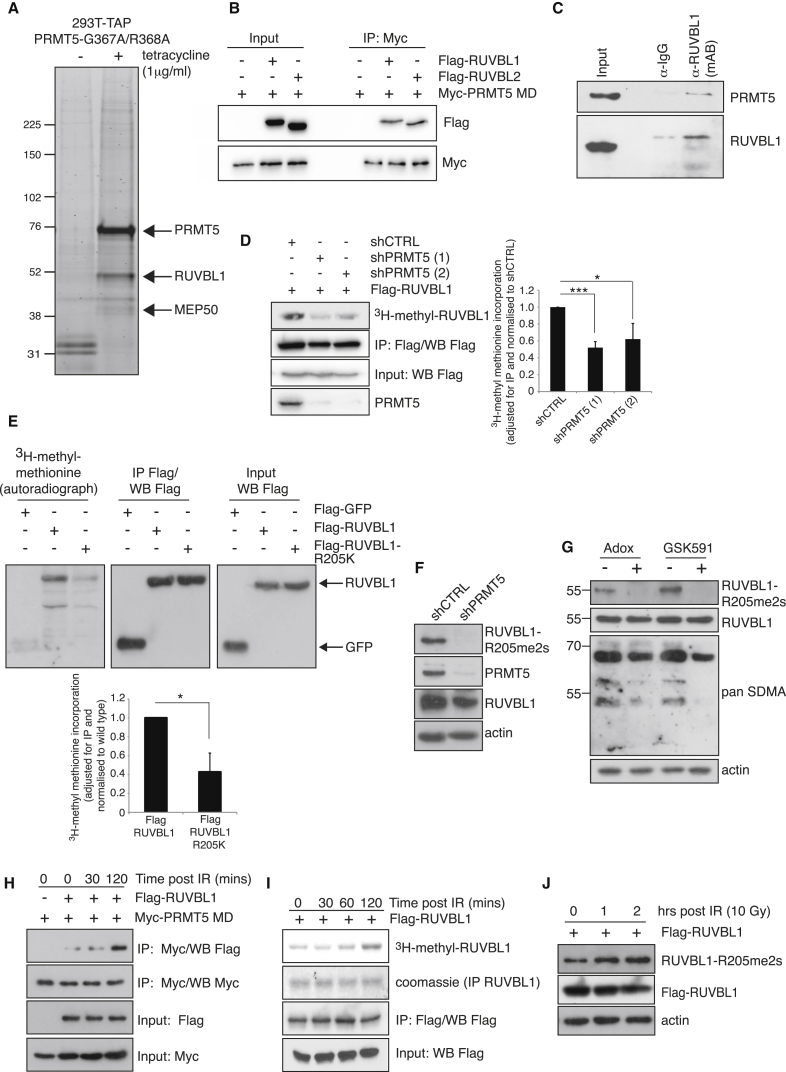
PRMT5 Binds to and Methylates RUVBL1 at R205 (A) SYPRO Ruby stain of PRMT5 protein complexes isolated from 293T cells stably expressing tetracycline-regulated TAP-tagged catalytically inactive PRMT5 (PRMT5 MD) is shown. (B) Co-immunoprecipitation of myc-PRMT5 MD with FLAG-tagged RUVBL1/RUVBL2 is shown. (C) Endogenous immunoprecipitation of RUVBL1 from HeLa cells co-immunoprecipitates endogenous PRMT5. (D) Transfected HeLa-shCTRL or shPRMT5 cell lines were treated with CHX/CAM and labeled with [^3^H]-methyl methionine. FLAG-RUVBL1 was immunoprecipitated, and incorporated methyl groups were detected by SDS-PAGE followed by autoradiograph. Quantification of [^3^H]-methyl signal intensity adjusted for immunoprecipitation (IP) and normalized to shCTRL is shown (mean ± SD; ^∗^p = 0.03 and ^∗∗∗^p = 0.0009). (E) Autoradiograph and immunoblots of 293T cells transfected with FLAG-RUVBL1 or FLAG-RUVBL1-R205K, treated with CHX/CAM and labeled with [^3^H]-methyl methionine. Quantification of [^3^H]-methyl signal intensity adjusted for IP and normalized to wild-type is shown (mean ± SD; n = 3). (F) Immunoblot analysis of endogenous RUVBL1-R205me2s in HeLa-shCTRL or shPRMT5 cell lines is shown. (G) Immunoblot analysis of endogenous RUVBL1-R205me2s in HeLa cells treated with Adox (100 μM; 24 hr) or GSK591 (5 μM; 24 hr) is shown. (H) Transfected 293T cells were damaged with 10Gy IR and myc-PRMT5 MD immunoprecipitated. Associated RUVBL1 was detected by FLAG immunoblotting. (I) Autoradiograph and immunoblots of 293T cells transfected with FLAG-RUVBL1, treated with CHX/CAM, and labeled with [^3^H]-methyl methionine for 4 hr before treating with 10Gy IR are shown. (J) Immunoblot analysis of whole-cell lysates transfected with FLAG-RUVBL1 and damaged with 10Gy IR. See also Figures S3–S5.

Having established that PRMT5 is an important component of HR-mediated DSB repair, we sought to identify PRMT5 substrates that could explain the mechanism by which this occurs. Enzyme-substrate interaction is often transient in nature; consequently, the identification of interactions can be particularly challenging. We therefore employed a substrate-trapping methodology using enzymatically inactive PRMT5 coupled with TAP-tag purification and mass spectrometry. While this approach enriched for a well-established PRMT5 cofactor (MEP50), it also identified the AAA+ ATPase RUVBL1 as a potential binding protein/substrate (Figure 3A). RUVBL1 and its binding partner RUVBL2 can form homohexameric assemblies (Matias et al., 2006, Petukhov et al., 2012) as well as a large dodecamer complex consisting of two hetero-hexameric rings with alternating RUVBL1 and RUVBL2 monomers (Gorynia et al., 2011, Lakomek et al., 2015). This protein complex predominantly functions as part of a number of high molecular weight nuclear complexes involved in chromatin remodeling, histone tail modification, and histone exchange. Importantly, RUVBL1-containing TIP60, SRCAP, and INO80 complexes are known to be involved in the DSB response (Alatwi and Downs, 2015, Dong et al., 2014, Gospodinov et al., 2011, Ikura et al., 2000, Jha et al., 2008, Murr et al., 2006, Sun et al., 2005, Tang et al., 2013, Wu et al., 2007). We therefore decided to further investigate the significance of the PRMT5/RUVBL1 interaction.

Ectopic expression of catalytically inactive myc-tagged PRMT5 in conjunction with FLAG-tagged RUVBL1 or RUVBL2 confirmed our initial mass spectrometry findings (Figure 3B). This was verified by reciprocal co-immunoprecipitation of endogenous RUVBL1 with endogenous PRMT5 (Figure 3C). We next examined the in vivo methylation status of RUVBL1 and/or RUVBL2 by labeling cells with radioactive [^3^H]-methyl methionine. The incorporation of radiolabeled methionine via de novo protein synthesis was efficiently blocked by the addition of cycloheximide and chloramphenicol, as indicated by the low levels of methyl-labeled GFP protein in FLAG-GFP-transfected cells (Figure S3A, lane 1). In cells, the [^3^H]-methyl methionine serves as a precursor for the in vivo synthesis of [^3^H]-SAM, which in turn functions as the essential methyl donor for all methylation reactions. Immunoprecipitated RUVBL1 and RUVBL2 exhibited substantial incorporation of the radioisotope (Figure S3A, top panel), indicating that both RUVBL1 and RUVBL2 are post-translationally modified via the addition of methyl groups in vivo. However, RUVBL1 and RUVBL2 also are methylated by the lysine methyltransferase G9a (Lee et al., 2010, Lee et al., 2011). We therefore repeated the in vivo [^3^H]-methyl methionine labeling using PRMT5-depleted cells, and we observed that the methylation of RUVBL1, but not RUVBL2, was significantly reduced by PRMT5 depletion (Figures 3D and S3B). Moreover, arginine methylation of RUVBL1 appeared to be specific to PRMT5, as recombinant PRMT1, the main mammalian asymmetric dimethyltransferase, failed to catalyze RUVBL1 methylation in vitro (Figure S3E).

PRMTs often direct methylation to arginine residues that reside within glycine-rich regions, the so-called RGG/RG motif (Thandapani et al., 2013). The RUVBL1 sequence contains three RG motifs, however, mutation of arginine to lysine within these sequences failed to prevent RUVBL1 methylation (data not shown). We therefore employed a non-biased mass spectrometry approach to facilitate residue identification. In five independent experiments, digestion with Asp-N consistently detected the presence of a methyl group on a single residue, R205 (Figure S3F). Residue R205 is highly conserved among higher and lower eukaryotes (Lakomek et al., 2015), implying functional significance. We therefore mutated R205 to lysine and validated our mass spectrometry finding of R205 as a site of methylation by in vivo [^3^H]-methyl methionine labeling of cells (Figure 3E). To verify that RUVBL1 is symmetrically dimethylated in cells, we generated a methyl-specific antibody (R205me2s). Methylation could be detected on FLAG-RUVBL1 wild-type, but not FLAG-RUVBL1-R205K, implying antibody specificity (Figure S3D). More importantly, endogenous RUVBL1-R205me2s was substantially reduced by treating cells with the pan-methyltransferase inhibitor Adox, by PRMT5 depletion, or through specific inhibition of PRMT5 activity with GSK591 (Chan-Penebre et al., 2015) (Figures 3F and 3G). Thus, RUVBL1-R205me2s occurs on endogenous RUVBL1 in a PRMT5-dependent manner. Although we were unable to show that PRMT5 directly catalyzes the dimethylation of RUVBL1 in vitro using recombinant proteins (data not shown), our observations that both the interaction of PRMT5 with RUVBL1 and methylation at R205 increase following exposure to IR (Figures 3H–3J), coupled with our comprehensive analysis using the RUVBL1-R205me2s antibody, strongly suggest that RUVBL1 is a direct substrate of PRMT5.

Strikingly, in silico analysis of R205 positioning using the solved crystal structure of hexameric RUVBL1 and the hetero-hexameric RUVBL1/RUVBL2 complex (Gorynia et al., 2011, Matias et al., 2006) designates R205 as surface facing within domain II, hence protruding from the hetero-hexameric ring (Figure S4A). Domain II has been proposed as a protein-protein interaction domain modulating the activity rather than the formation of the RUVBL1/RUVBL2 hetero-hexameric complex (Gorynia et al., 2011, López-Perrote et al., 2012, Matias et al., 2006). In agreement with this, ectopic expression of RUVBL1-R205K had no effect on the ability of RUVBL1 to co-immunoprecipitate RUVBL2 (Figure S5A). Importantly, these findings demonstrate that the R205-to-lysine conservative mutation does not disrupt the fold of monomeric RUVBL1 or its ability to interact with its binding cofactor, RUVBL2.

### RUVBL1-R205 Methylation Is a Critical Component of HR-Mediated DSB Repair

**Figure 4:**
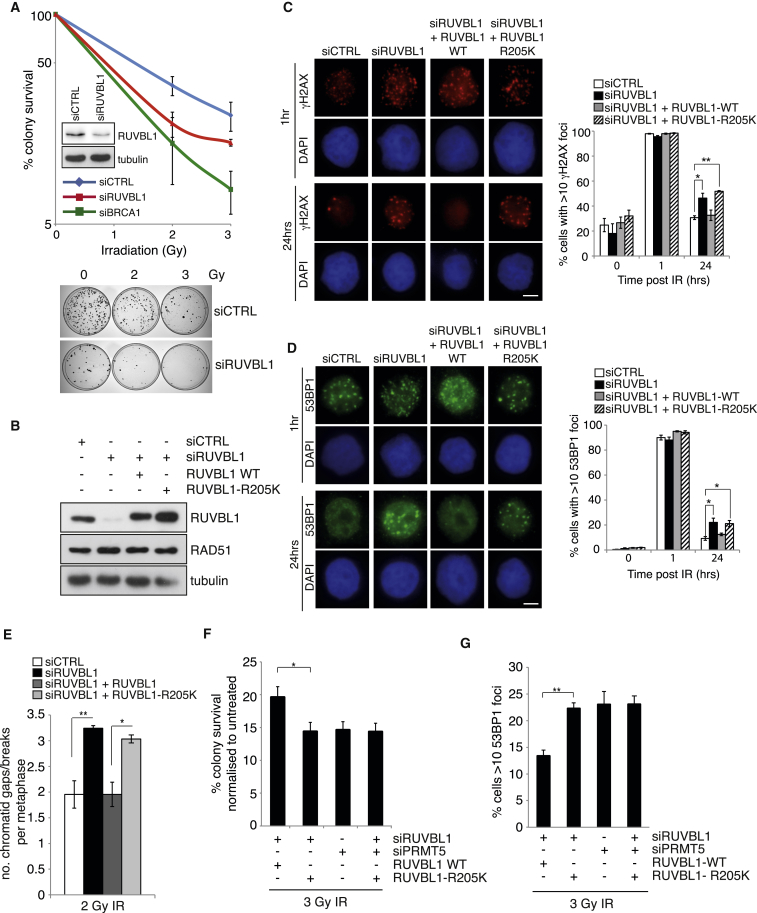
Mutation of RUVBL1 at R205 Impedes DSB Repair after Ionizing Radiation (A) Cells were exposed to increasing doses of IR and cell viability was determined by colony assay. Representative images are displayed under quantification, and the inset immunoblot depicts RUVBL1 knockdown (mean ± SD; n = 3). (B) Generation of siRNA-resistant RUVBL1 wild-type (WT) or RUVBL1 methyl-deficient (R205K) HeLa cell lines is shown. (C and D) Cells were transfected with siRUVBL1, exposed to 3Gy IR, and the number of cells with >10 γH2AX (C) or 53BP1 (D) foci were quantified (mean ± SEM; n = 3). (E) Quantification of chromatid gaps and breaks after 2Gy IR is shown (mean ± SEM; n = 3). (F and G) HeLa-RUVBL1 WT or HeLa-RUVBL1-R205K cells were transfected with the indicated siRNAs and exposed to 3Gy IR. Cell survival was assessed by colony assay (F) (mean ± SD; n = 3). Number of cells with >10 53BP1 foci was scored (G) (mean ± SEM; ^∗∗^p = 0.0095; n = 3). Scale bar, 10 μm. See also Figure S5.

Several lines of evidence have implicated the importance of RUVBL1 as a regulator of the DNA damage response (Gospodinov et al., 2009, Jha et al., 2008, Tang et al., 2013). To verify that RUVBL1 plays a role in the DSB repair pathway, we carried out colony survival assays using RUVBL1-depleted cells in combination with IR-induced DNA damage. In a manner similar to PRMT5 depletion (Figure 1I), silencing of RUVBL1 rendered cells hypersensitive to IR-induced DNA damage (Figure 4A). In keeping with hypersensitivity of RUVBL1-depleted cells to IR, silencing RUVBL1 expression hindered the clearance of γH2AX and 53BP1 foci at 24 hr post-irradiation, implying defective repair (Figure 4C). Since both PRMT5 and RUVBL1 are necessary for an effective DNA damage response after IR and given that PRMT5 is required for the methylation of RUVBL1 at R205, we hypothesized that arginine methylation of RUVBL1 could be an important regulatory event during HR. To address this, we generated cell lines stably expressing wild-type (WT) or methyl-deficient RUVBL1 (RUVBL1-R205K) that were resistant to siRNA to allow the depletion of endogenous, but not exogenous, RUVBL1 (Figure 4B). Importantly, reconstitution of RUVBL1-depleted cells with wild-type RUVBL1, but not the methyl-defective mutant, completely restored γH2AX and 53BP1 foci clearance (Figures 4C and 4D). Moreover, methyl-deficient RUVBL1 demonstrated increased retention of 53BP1 at early time points (4 hr) (Figure S5B). Together, these findings suggest that PRMT5-dependent methylation of RUVBL1 is essential for its role in promoting DSB repair. In agreement with this, the increase in unrepaired chromatid gaps/breaks exhibited by cells depleted of endogenous RUVBL1 was completely restored by ectopic expression of wild-type RUVBL1, but not the R205K mutant (Figure 4E). Significantly, the cellular hypersensitivity to IR and defective resolution of 53BP1 foci from the loss of RUVBL1-R205 methylation were completely epistatic with a loss of PRMT5, indicating that the function of PRMT5 in promoting the repair of DNA DSBs after IR is primarily mediated through its ability to methylate RUVBL1 (Figures 4F and 4G). Taken together, these data indicate that methylation of RUVBL1 at R205 by PRMT5 is a critical post-translational modification required for regulation of the cellular DNA damage response invoked by DSBs.

**Figure 5:**
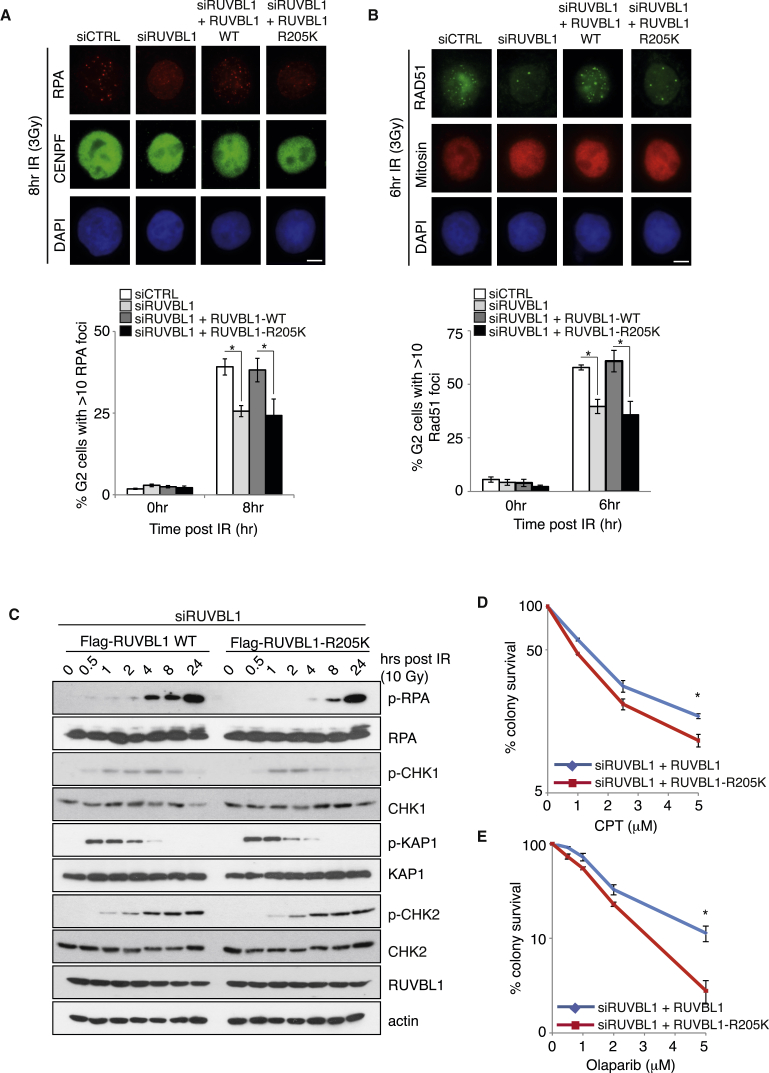
Methylation of RUVBL1 Is Required for HR, but Not ATM Signaling (A and B) HeLa-RUVBL1-WT and HeLa-RUVBL1-R205K cells were transfected with siRUVBL1, exposed to 3Gy IR, and harvested at the time points indicated. CENPF/mitosin-positive cells were scored for RPA (A) and RAD51 (B) foci formation (mean ± SEM; n = 3). (C) HeLa-RUVBL1-WT and HeLa-RUVBL1-R205K cells were transfected with siRUVBL1, exposed to 10Gy IR, and harvested at the time points indicated for immunoblot analysis. (D and E) HeLa-RUVBL1-WT and HeLa-RUVBL1-R205K cells were transfected with siRUVBL1, treated with increasing doses of CPT (D) or Olaparib (E), and survival was assessed by colony survival assay (mean ± SEM; n = 3). Scale bar, 10 μm. See also Figure S5.

To investigate this further, we used our RUVBL1 siRNA knockdown/complementation system (Figure 4B) to determine whether PRMT5-dependent methylation of RUVBL1 plays a role in DSB resection. Compared to control cells, silencing of RUVBL1 greatly reduced the appearance of RPA foci, which could be restored by ectopic expression of wild-type, but not R205K, RUVBL1 (Figure 5A), suggesting that methylation of RUVBL1 is critical for the generation of regions of ssDNA at sites of DSBs. Consistent with this, silencing RUVBL1 also reduced the formation of IR-induced RAD51 foci, which again could be rescued by wild-type, but not R205K, RUVBL1 (Figure 5B). Finally, loss of RUVBL1 methylation on R205 led to a similar delay in RPA phosphorylation in response to IR, without affecting the ability of ATM to be activated, and an increased sensitivity to CPT and Olaparib comparable to PRMT5-depleted cells (Figures 5C–5E and S5C). Collectively, these data demonstrate that PRMT5 promotes HR-mediated repair of DSBs through its ability to regulate the methylation of RUVBL1 on R205.

### PRMT5-Dependent Methylation of RUVBL1-R205 Regulates TIP60 HAT Activity and HR through the Removal of 53BP1

**Figure 6:**
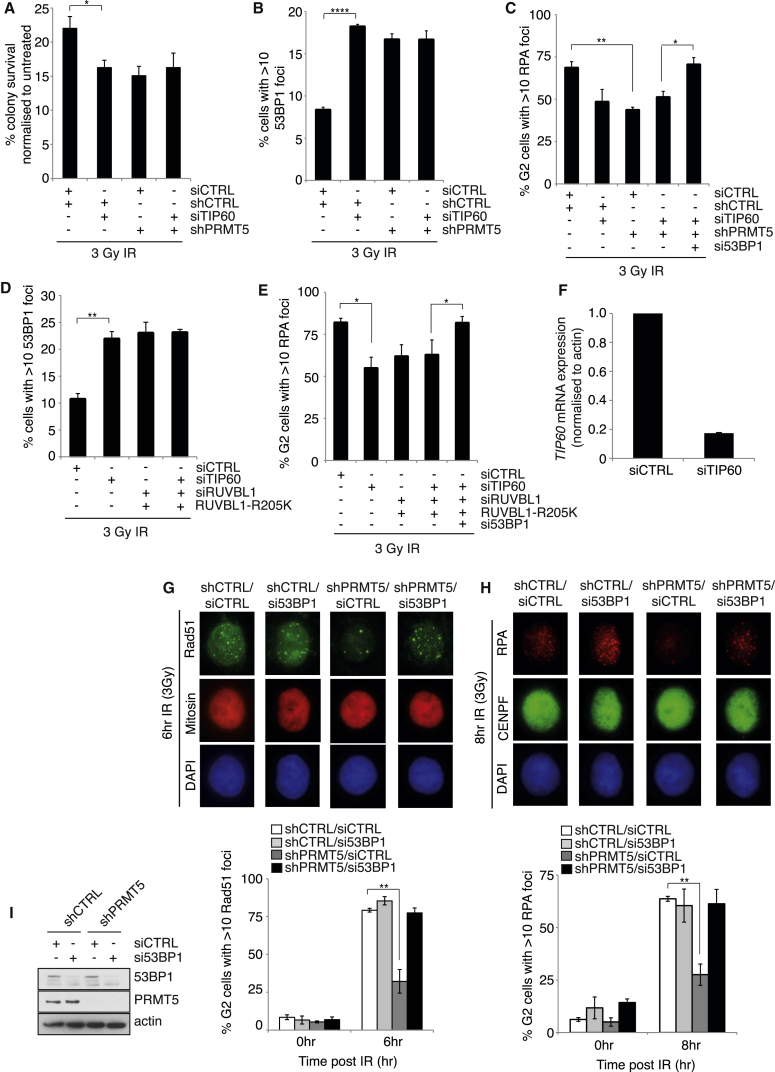
PRMT5-Dependent Methylation of RUVBL1 at R205 Regulates 53BP1 Removal and Is Epistatic with TIP60 (A) HeLa-shCTRL or shPRMT5 cells were siRNA transfected, exposed to 3Gy IR, and cell viability was assessed by colony survival. Results are expressed normalized to the respective non-treatment control (mean ± SD; n = 3). (B and C) HeLa-shCTRL or shPRMT5 cells were siRNA transfected, exposed to 3Gy IR, and stained for 53BP1 (B) or CENPF/RPA (C) foci 24 and 8 hr after damage, respectively (mean ± SEM; n = 3). (D and E) HeLa-shCTRL or RUVBL1-R205K cells were siRNA transfected, exposed to 3Gy IR, and stained for 53BP1 (D) or CENPF/RPA (E) foci 24 and 8 hr after damage, respectively (mean ± SEM; n = 3). (F) Validation of siTIP60 knockdown is shown. (G) Cells were transfected with si53BP1 and the number of mitosin-positive cells with >10 RAD51 foci was quantified (mean ± SEM; n = 3). (H) Cells were transfected with si53BP1 and the number of CENPF-positive cells with >10 RPA foci was quantified (mean ± SEM; n = 3). (I) Validation of 53BP1 siRNA SMARTpool. See also Figure S6.

RUVBL1 is an integral component of the TIP60, INO80, and SRCAP complexes, all of which have been implicated in various DNA repair processes (Alatwi and Downs, 2015, Dong et al., 2014, Gospodinov et al., 2011, Ikura et al., 2000, Jha et al., 2008, Murr et al., 2006, Sun et al., 2005, Tang et al., 2013, Wu et al., 2007). We therefore performed epistasis analysis to dissect out which RUVBL1-containing complex was regulated in a PRMT5-dependent manner. Interestingly, increased sensitivity of PRMT5-depleted cells to IR, retention of 53BP1 foci, and defective RPA recruitment were epistatic with the loss of TIP60 (Figures 6A–6C and 6F). In contrast, co-depletion of PRMT5 and SRCAP resulted in a synergistic reduction in RPA recruitment (Figure S6A), while depletion of INO80 resulted in defective recruitment of 53BP1 at early time points after damage, a phenotype not exhibited after PRMT5 depletion (Figure S6B). These observations suggest that the methylation of RUVBL1 by PRMT5 is specifically targeted to complexes containing TIP60 and not SRCAP or INO80. Consistent with this, the DSB end resection defect observed in cells expressing the methyl-deficient RUVBL1 was also epistatic with the loss of TIP60 (Figures 6D and 6E).

One mechanism by which the TIP60 acetyltransferase complex has been implicated in regulating HR is through its ability to modulate the retention of 53BP1 at sites of DSBs; TIP60-mediated acetylation of histone H4K16 blocks the Tudor domain of 53BP1 from binding to methylated H4K20 (Tang et al., 2013). Since RUVBL1 is an essential TIP60 cofactor, allowing proper assembly and catalytic activity of the complex (Jha et al., 2008, Jha et al., 2013), and both PRMT5 and RUVBL1-R205K are epistatic with TIP60 for 53BP1 mobilization (Figures 6B and 6D), we hypothesized that the failure of cells lacking PRMT5 or RUVBL1 methylation to efficiently resect DSBs could arise as a consequence of an inability to remove the inhibitory effect of 53BP1. To investigate this, we co-depleted 53BP1 in cells stably depleted of PRMT5, and we evaluated RAD51 and RPA localization after IR in late S/G2 cells. Strikingly, the depletion of 53BP1 completely restored the resection defect resulting from PRMT5 depletion, allowing RAD51 and RPA localization comparable to that of control cells (Figures 6G–6I). Indeed, the finding that further co-depletion of 53BP1 could restore the epistatic defect in RPA foci formation (Figures 6C and 6E) supports our hypothesis that PRMT5 and methylated RUVBL1 are functioning in the same pathway as TIP60, which is required for effective 53BP1 mobilization from DNA break ends.

**Figure 7:**
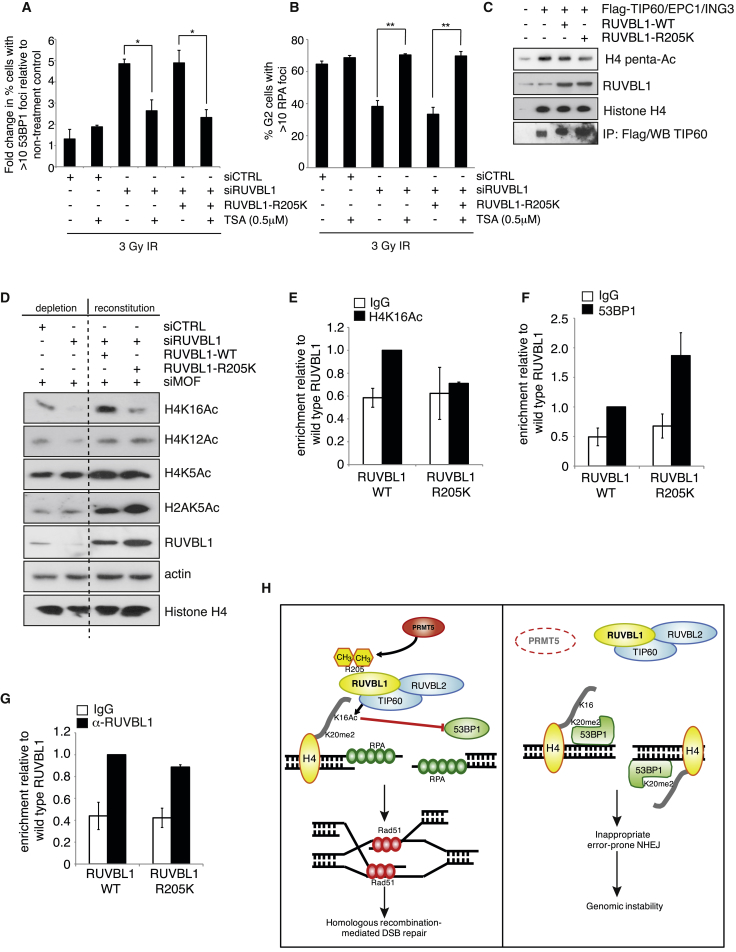
PRMT5-Dependent Methylation of RUVBL1 Regulates TIP60 Acetyltransferase Activity (A and B) HeLa shCTRL or RUVBL1-R205K cells were siRNA transfected and treated with TSA (0.5 μM) for 16 hr before exposure to 3Gy IR. The number of cells with >10 53BP1 foci 24 hr after damage (A) and the number of CENPF-positive cells with >10 RPA foci 8 hr after damage (B) were quantified (mean ± SEM; n = 3). (C) In vitro TIP60 histone acetyltransferase assay. 293T cells were transfected with the TIP60 piccolo complex (TIP60/EPC1/ING3) and wild-type or methyl-deficient RUVBL1. TIP60 activity was determined by immunoblotting for recombinant H4 acetylation. (D) HeLa shCTRL, RUVBL1-WT, or -R205K cells were siRNA transfected, and acetylated histone marks were determined by immunoblotting. (E–G) ChIP performed with H4K16Ac (E), 53BP1 (F), and RUVBL1 (G) antibodies in the presence of mCherry-Lac1-Fok1-DD-induced DSBs, in U20S-265-RUVBL1 wild-type or RUVBL1-R205K-expressing cells. Data represent the average percentage input across the four primer pairs expressed relative to RUVBL1 WT (mean ± SEM; n = 3). (H) Model of how PRMT5-dependent methylation of RUVBL1 impacts on HR repair. PRMT5 symmetrically dimethylates RUVBL1 at R205, which stimulates TIP60 HAT activity enabling H4K16 acetylation and the removal of 53BP1 from DNA breaks. After end resection, RPA loading, and subsequent displacement by RAD51, homology searching and strand invasion can proceed enabling error-free HR-mediated repair of DSBs. Removal of PRMT5 results in hypomethylated RUVBL1 and ineffective TIP60 activity, causing reduced H4K16 acetylation and 53BP1 retention. Consequently, end resectioning is greatly impaired during late S/G2 phase, resulting in defective RPA and RAD51 recruitment, inappropriate error-prone NHEJ, and genomic instability. See also Figures S6 and S7.

Based on these findings, we rationalized that if methylation of RUVBL1 is required for histone acetylation, inhibiting histone deacetylases (HDACs) should restore the defect in 53BP1 mobilization and RPA foci formation. Consistent with this premise, exposure of cells expressing the RUVBL1-R205K mutant to Trichostatin A (TSA) rescued the defect in 53BP1 mobilization and RPA foci formation after IR (Figures 7A and 7B). Given that TIP60 is required to evict 53BP1 from chromatin surrounding a DSB through its ability to acetylate K16 on histone H4 (Tang et al., 2013), we postulated that the methylation of RUVBL1 may be required for this activity. In keeping with this, we observed that the expression of methyl-deficient RUVBL1 reduced the HAT activity of TIP60 in vitro (Figure 7C), and, more importantly, this resulted in a reduction of H4K16 acetylation following the depletion of MOF, which alongside TIP60 is the principle HAT that catalyzes this histone modification (Figures 7D and S6D). Interestingly, loss of RUVBL1 methylation did not affect the TIP60-dependent acetylation of H4 on K5 and K12 or histone H2A on K5, indicating that this post-translational modification of RUVBL1 is specific to H4K16 acetylation.

To strengthen our hypothesis that the increased presence of 53BP1 at sites of DSBs in cells lacking PRMT5 or expressing methyl-deficient RUVBL1 is due to a failure to remove 53BP1 from the surrounding chromatin, rather than merely being a marker of unrepaired DNA breaks, we utilized the mCherry-LacI-FokI nuclease system to create a DSB within a single genomic locus, enabling the direct quantification of chromatin-bound 53BP1 through chromatin immunoprecipitation (ChIP). In this system, treatment of control cells with 4-hydroxy-tamoxifen (4-OHT) and the Shield-1 ligand induces DNA damage, as evident by a single 53BP1 focus, and an enrichment of H4K16 acetylation at break ends (Figures S7A and S7B) (Shanbhag et al., 2010, Tang et al., 2013). In contrast, PRMT5 depletion or expression of methyl-deficient RUVBL1 suppressed H4K16 acetylation while promoting 53BP1 retention (Figures 7E, 7F, and S7C–S7E). Interestingly, both wild-type and methyl-deficient RUVBL1 were effectively recruited to DSB ends, suggesting that arginine methylation does not regulate TIP60 chromatin association (Figure 7G). Collectively, these findings highlight the importance of arginine methylation as a mechanism to control DNA repair pathway choice via its ability to regulate the enzymatic activity of TIP60 and H4K16 acetylation at DSB ends.

## Discussion

While the critical role of protein modifications in the control of the DNA damage response is becoming increasingly apparent, the majority of studies have focused on how this is regulated by phosphorylation, ubiquitylation, sumoylation, and lysine methylation. In this study, we have demonstrated the importance of the symmetric arginine methyltransferase, PRMT5, for homologous recombination-mediated repair and the maintenance of genomic stability. Critically, we have discovered that PRMT5 regulates the methylation of RUVBL1 and that this event is crucial for the coordination of RUVBL1-mediated DSB repair activity by promoting TIP60 activity and H4K16 acetylation. Consequently, cells depleted of PRMT5, or expressing a form of RUVBL1 that cannot be methylated, are unable to efficiently undergo DSB end resection, are defective in HR, and display increased genome instability when treated with genotoxic agents (Figure 7H). Given that the DNA repair response defects in cells lacking PRMT5 or RUVBL1 methylation can be reversed by the concomitant depletion of 53BP1, our data suggest that arginine methylation is a critical determinant for regulating the decision of whether a DSB is channeled into an NHEJ- or HR-dependent repair pathway.

The role of PRMT5 in the DNA damage response has been largely unexplored, with only three known DNA repair substrates, p53, Fen1, and Rad9 (Guo et al., 2010, He et al., 2011, Jansson et al., 2008). However, while these studies addressed the consequence of expressing a methyl-deficient form of these proteins, none investigated the significance of PRMT5 per se during the DNA damage response. Our comprehensive analysis clearly demonstrates a critical role for PRMT5 during HR-mediated DSB repair. Interestingly, we observed elevated levels of spontaneous DSBs, as measured by γH2AX/53BP1 foci after genetic deletion of PRMT5 in primary, non-immortalized MEFs (Figures 1F–1H), implying a role for PRMT5 in maintaining genome stability, potentially in response to endogenous damage such as replication fork stalling and collapse. While this could potentially be mediated by Fen1 and/or Rad9 methylation, RUVBL1, TIP60, and H4K16 acetylation also have been implicated in the repair of DNA interstrand crosslinks (Rajendra et al., 2014, Renaud et al., 2016). This suggests that the identified mechanism of DNA end resection involving the methylation of RUVBL1 may be required for the effective processing of different types of endogenous replication-associated DNA lesions.

While we were unable to show direct methylation of RUVBL1 by PRMT5 in vitro, it is clear that PRMT5 is required for in vivo RUVBL1 methylation, and this implies that PRMT5-dependent methylation of RUVBL1 in cells is more complex than a simple enzyme-substrate interaction. Very little is known about the regulation of PRMT5; however, our finding that RUVBL1 methylation is enhanced post-IR implicates dynamic regulation downstream of the initial DSB response. Understanding this could reveal important insights into how PRMT5 activity is controlled in a temporal and spatial manner.

Our observation that R205 of RUVBL1 is methylated by PRMT5, but not the corresponding residue in RUVBL2 (R206), which is conserved both at the sequence and structural levels, highlights an interesting divergent function between these two highly related proteins. Recent insights into the solved hetero-hexameric structure of *chaetomium thermophilum* (*ct*) full-length RUVBL1/RUVBL2 complex revealed that the domain II (DII) region, containing R205 in human RUVBL1 and its equivalent R206 in human RUVBL2, can adopt widely different orientations with respect to the ATPase core (Lakomek et al., 2015), confirming the flexibility originally inferred from the homohexameric RUVBL1 structure (Matias et al., 2006). In human and ct RUVBL1, R205 is exposed to the solvent and is therefore accessible to PRMT5 for methylation (Figure S4A). In contrast, in the ADP-bound form of ct RUVBL2, R206 is found somewhat buried and capped by the side chain of Met187 (Figures S4B and S4C). This suggests that R205 of RUVBL1 has evolved to provide a regulatory function to RUVBL1/2-containing protein complexes.

While RUVBL1 is an integral component of the TIP60, INO80, and SRCAP complexes, all of which have been implicated in various stages of the DSB and HR-mediated repair (Alatwi and Downs, 2015, Dong et al., 2014, Gospodinov et al., 2011, Ikura et al., 2000, Jha et al., 2008, Jha and Dutta, 2009, Murr et al., 2006, Sun et al., 2005, Tang et al., 2013, Wu et al., 2007), our findings identify a specific function for RUVBL1 methylation in regulating the activity of the TIP60 complex. Moreover, the TIP60 complex also is known to have multiple roles during the DNA damage response. Surprisingly, very little is understood about how specific TIP60 activities are controlled in a temporal and spatial manner. Here we have shown that methylated RUVBL1 is an important regulator of a subset of TIP60 activities, namely H4K16 acetylation and 53BP1 eviction, and not others, such as ATM activation and H2A.Z mobilization (Figure S6C) (Choi et al., 2009, Kusch et al., 2004). The underlying reasons for this are unclear. However, interestingly, the location of R205 within DII could be of significance, as DII contains an OB fold proposed to act as a nucleotide/protein-binding interface; thus, one potential mechanism by which methylation of RUVBL1 could be modulating TIP60 activity is by altering the binding of specific cofactors. Indeed, while methylation does not change the overall charge of the residue, it does alter the availability of hydrogen donors and increases van der Waals interactive forces (Liu et al., 2010, Yang et al., 2010). Despite the fact that methylated residues are principally read by Tudor domain-containing proteins, none of the known components of the human TIP60 complex possesses a Tudor domain. This raises the intriguing possibility that additional regulatory components of the TIP60 complex may exist, which can specifically bind to methylated arginine residues using alternative protein domains. Indeed, an important constituent of the DNA damage TIP60 complex is TRRAP. TRRAP is not required for the activation of ATM or its downstream signaling cascade, but it is required for chromatin recruitment of TIP60, histone H4 acetylation, chromatin relaxation, and loading of RAD51 to sites of DNA damage (Murr et al., 2006). Since these observations are remarkably similar to those we have observed for methylated RUVBL1, it would be interesting to ascertain if the methylation of RUVBL1 modulates TRRAP incorporation or function.

In summary, our findings reveal the importance of arginine methylation in the temporal and spatial regulation of the TIP60 complex, coordinating substrate specificity and activity. Our study further supports the notion that regulation of events at chromatin after DNA damage is exquisitely controlled through a combination of protein complex recruitment and post-translational modification and highlights the importance of crosstalk between arginine methylation and histone acetylation for appropriate DNA repair pathway choice and HR-mediated repair.

## STAR★Methods

### Key Resources Table

**Table:** 

REAGENT or RESOURCE	SOURCE	IDENTIFIER
**Antibodies**		

H2AX	Millipore	05-636
53BP1	Millipore	MAB3802
PRMT5	Millipore	07-405
PRMT5	Cell Signaling Technologies	2252
Actin	Sigma	A2228
Tubulin	Sigma	T6199
Mitosin	BD	610768
Rad51	Millipore	PC130
RPA (34-19)	Calbiochem	NA18
CENPF	Abcam	AB5
P-ATM (ser1981)	R&D Systems	AF1655
ATM	GeneTex	GTX70103
P-RPA (ser 4/8)	Bethyl	A300-245A
P-Chk1 (ser 345)	Cell Signaling Technologies	2348
Chk1	Santa Cruz	SC8408
P-Chk2 (T68)	Abcam	Ab32148
Chk2	Steve Elledge	N/A
Symmetric Dimethyl Arginine	Cell Signaling Technologies	13222
P-KAP1	Bethyl	A300-767-AM
KAP1	Bethyl	A300-274-AM
Flag-HRP	Cell Signaling Technologies	2044
RUVBL1	Thermo Scientific	PA5-29278
RUVBL1	Sigma	SAB4200194
RUVBL2	Thermo Scientific	PA5-29871
GFP	Roche	11 814 460 001
c-myc (9E10)	Santa Cruz	SC-40
H4 Penta Acetyl	Millipore	06-946
Histone H4	Abcam	AB7311
Histone H4	Bethyl	A2300-647A-T
Tip60	Millipore	07-038
H4K16Ac	Bryan Turner	N/A
H4K5Ac	Bryan Turner	N/A
H4K8Ac	Bryan Turner	N/A
H2AK5Ac	Bryan Turner	N/A
H4K16Ac	Abcam	AB109463
H4K16Ac	Millipore	07-329
H4K12Ac	Cell Signaling Technologies	13944
Anti-Rabbit IgG	Millipore	12370
Anti-Mouse IgG	Cell Signaling Technologies	54155
BRCA1	Santa Cruz	SC6954
Dylight Goat-Anti Rabbit 488	Thermo Scientific	35552
Alexa Fluor anti-mouse 594	Life Technologies	A11-032
Phospho Histone H3 (Ser10)	Cell Signaling Technologies	9701
H2A.Z	Abcam	AB4174
RUVBL1-R205me2s	This paper	
Anti-Rabbit IgG HRP	DAKO	P0399
Anti-mouse IgG HRP	DAKO	P0447

**Chemicals, Peptides, and Recombinant Proteins**		

4-OHT	Sigma	H7904
Olaparib	Selleck Chemicals	S1060
Camptothecin	Sigma	C9911
Interferin	PolyPlus	409-10
JetPei	PolyPlus	101-10N
Lipofectamine 2000	Thermo Fisher	11668027
Tetracycline	Sigma	87128
Methylene Blue	Sigma	M9140
Trichostatin A (TSA)	Sigma	T8552
Progold Antifade Mounting Reagent (With DAPI)	Life Technologies	P36935
KaryoMax Colcemid	GIBCO	15212-012
Poly-D-Lysine	Sigma	P7886
Giemsa Staining Solution	Sigma	GS500
Propidium Ioidide	Sigma	P4170
Aphidicolin	Sigma	A4487
Cycloheximide	Sigma	01810
Chloramphenicol	Sigma	C0378
IgG Sepharose 6 fast flow	GE Healthcare	GE17-0969-01
Calmodulin resin	Agilent	214303
Protein G Sepharose	GE Healthcare	17-0618
Protein G Agarose	Roche	11719416001
anti-Flag M2-affinity beads	Sigma	A2220
TEV protease	Promega	V6101
SYPRO Ruby	Lonza	LZ50562
Recombinant Histone H4	NEB	M2504S
GSK591	SGC	N/A
Adox	Sigma	A7154
L-Methyl-3H methionine	Perkin Elmer	NET061X
S-[methyl-^3^H]–adenosyl-L-methionine	Perkin Elmer	NET155H
EN3HANCE	Perkin Elmer	6NE970C
Shield-1	Clontech	632189

**Experimental Models: Cell Lines**		

U20S HR reporter cell lines	Professor J. Stark (City of Good Hope Hospital, CA) Gunn and Stark, 2012	
U20S-265 cell line	Professor Roger Greenberg (University of Pennsylvania) Tang et al., 2013	
HeLa	ATCC	CCL-2
293T	ATCC	CRL-3216

**Experimental Models: Organisms/Strains**		

Mouse: Prmt5^tm1a(EUCOMM)Wtsi^	European Mouse Mutant Archive (EMMA)	MGI:4432546
Mouse: B6.129-Gt(ROSA)26Sortm1(cre/ERT2)Tyj/J	The Jackson Laboratory	008463

**Recombinant DNA**		

pCMV2B-Flag-RUVBL1-R205K	This paper	
pHIV-zsGreen-Flag-RUVBL1-siRNA resistant	This paper	
pHIV-zsGreen-Flag-RUVBL1-siRNA resistant-R205K	This paper	
pNIC28-Bsa4-6X His-TEV RUVBL1	Dr. Walid Houry (University of Toronto, Canada)	
p11-6X His-TEV RUVBL2	Dr. Walid Houry (University of Toronto, Canada)	
pLKO.1-TRC (plasmid no. 10878)	Dr. David Root (Moffat et al., 2006)	Addgene
pLKO.1-TRC-shPRMT5 (1)	This paper	
pLKO.1-TRC-shPRMT5 (2)	This paper	
pcDNA 4/TO-C-TAP-PRMT5 G367A/R368A	This paper	

**Sequence-Based Reagents**		

siRUVBL1 (5′-GUUUACUCAACUGAGAUCA-3′)	Broad Institute GPP Web Portal	http://portals.broadinstitute.org/gpp/public
siTIP60 (5′-CCUCAAUCUCAUCAACUAC-3′)	Jeong et al., 2011	
siPRMT5 (5′- CGAAAUAGCUGACACACUA-3′)	Broad Institute GPP Web Portal	http://portals.broadinstitute.org/gpp/public
siSRCAP (5′- CGGAUAGACAUGGGUCGAUUU-3′)	Broad Institute GPP Web Portal	http://portals.broadinstitute.org/gpp/public
shCTRL (5′ CCTAAGGTTAAGTCGCCCTCG-3′)	Sarbassov et al., 2005	
shPRMT5 (1) (5′-GCGTTTCAAGAGGGAGTTCAT-3)	Broad Institute GPP Web Portal	http://portals.broadinstitute.org/gpp/public
shPRMT5 (2) (5′-AGGGACTGGAAT ACGCTAATT-3′)	Broad Institute GPP Web Portal	http://portals.broadinstitute.org/gpp/public
siBRCA1 (5′-GCUCCUCUCACUCUUCAGU-3′)	In house design	
si53BP1	Dharmacon	L-003548-00
siINO80	Dharmacon	L-003548-00
siBRCA2	Dharmacon	L-003462
siMOF	Dharmacon	L-014800-02
MISSON siRNA Universal Negative Control No.1	Sigma	SIC001

**Software and Algorithms**		

Scaffold	Proteome Software	http://www.proteomesoftware.com/products/scaffold
Velocity Software (V4.1)	Improvision, Perkin Elmer	
PDB2PQR for electrostatic interactions	Dolinsky et al., 2004	http://www.poissonboltzmann.org
APBS for electrostatic interactions	Baker et al., 2001	http://www.poissonboltzmann.org

**Other**		

### Contact for Reagent and Resource Sharing

Further information and requests for reagents may be directed to the corresponding author, Dr. Clare Davies (c.c.davies@bham.ac.uk).

### Experimental Models and Subject Details

#### Mice

*prmt5*^f/f^;CreERT2 mouse embryonic fibroblasts (MEFs) were generated by breeding *prmt5*^f/f^ and CreERT2^tg/+^ mice. Embryos were harvested at embryonic day 13.5 (E13.5) as described previously (Nilausen and Green, 1965). Gene deletion of *Prmt5* in cultured *prmt5*^f/f^;CreERT2 MEFs was induced by a 24 hr treatment of 500nM 4-hydroxy-tamoxifen (4-OHT; Sigma Aldrich). All experiments were conducted 120 hr later to ensure complete gene deletion. Animals were maintained in a pathogen-free facility at the University of Birmingham, and all animal experiments were carried out under license in accordance with the UK Home Office Animals (Scientific Procedures) Act (1986) and institutional guidelines.

#### Cell Culture and Cell Lines

HeLa, Human Embryonic Kidney 293T cells (HEK293T), U2OS HR reporter cells and U2OS-265 (Tang et al., 2013) were grown in Dulbecco’s modified Eagle’s medium (DMEM) supplemented with 10% fetal bovine serum (FBS) (GIBCO) and penicillin/streptomycin (Sigma Aldrich). Mouse Embryonic Fibroblasts were grown in Dulbecco’s modified Eagle’s medium supplemented with 10% heat inactivated fetal bovine serum (FBS) (GIBCO) and penicillin/streptomycin. U20S HR reporter cell lines were obtained from Professor Jeremy Stark (City of Good Hope Hospital, CA, USA), U20S-265 from Professor Roger Greenberg (Perelman School of Medicine, University of Pennsylvania, USA).

### Methods Details

#### siRNA Transfections and Establishment of Stable Cell Lines

siRNA sequences were synthesized by Sigma unless otherwise stated. siRNA transfections (30nM – 160nM/transfection) were carried out using Interferin (Polyplus) according to the manufacturer’s instructions, with total siRNA not exceeding 160nM. All experiments were performed at least 48 hr after knockdown. See Key Resource Table for siRNA sequences. siRNA resistant Flag-tagged RUVBL1 constructs were generated by mutating the sequence complementary to siRUVBL1 from GTT-TAC-TCA-ACT-GAG-ATC-A to GT**C**-TA**T**-**AGC**-ACT-GAG-ATC-A using the Q5 site-directed mutagenesis kit (NEB). To generate stable PRMT5-depleted cells, shPRMT5 sequences were obtained from the Broad Institute GPP Web Portal and cloned into pLKO.1-TRC, followed by selection with 1 μg/ml puromycin. All experiments were conducted on low passage cells to minimize effects of chronic PRMT5 depletion. To enable expression of ectopic RUVBL1 WT or R205K with concurrent depletion of endogenous RUVBL1, HeLa cells were lentivirally infected with pHIV-zsGreen-Flag-RUVBL1-siRNA resistant constructs. GFP positive populations were isolated by flow cytometry.

#### Colony Survival Assays

Cells were plated at low density 24 hr prior to treatment with increasing doses of ionizing radiation, Camptothecin (Sigma) or Olaparib (Selleck Chemicals). Cell densities were adjusted in accordance with increasing dose of damaging agent. Cells were treated with Camptothecin for 4 hr before being washed once in PBS followed by supplementation with fresh complete media. For Olaparib treatment, drug was supplemented to media every three days. Colonies were fixed and stained after 14 days with 2% methylene blue (Sigma Aldrich) in 50% ethanol. Data expressed is normalized to respective non-treatment control.

#### Metaphase Spreads

Chromosomal aberrations were scored in Giemsa stained metaphase spreads. For chromosome aberrations, demecolcine (Sigma) was added 3-4 hr prior to harvesting at a final concentration of 0.2 μg/ml. Cells were harvested by trypsinization, subjected to hypotonic shock for 1 hr at 37°C in 0.3M sodium citrate and fixed in 3:1 methanol:acetic acid solution. Cells were dropped onto acetic acid humidified slides, stained for 15 min in Giemsa-modified (Sigma) solution (5% v/v in H_2_O) and washed in water for 5 min.

#### Immunofluorescence, Microscopy and Image Analysis

Cells were plated on poly-D-Lysine coated coverslips (50 μg/ml) (Sigma Aldrich) 24 hr prior to treatment with ionising radiation. For HDAC inhibitor studies, cells were pretreated for 16 hr with TSA (0.5 μM) before 3Gy irradiation. Coverslips were placed into ice-cold pre-extraction buffer for 7 min (10mM PIPES pH 6.8, 300mM sucrose, 20mM NaCl, 3mM MgCl_2_ 0.5% Triton X-100), then fixed for 10 min in 4% PFA. After washing in phosphate-buffered saline (PBS) and 1 hr block (10% FCS in PBS), cells were incubated in primary antibody overnight at 4°C and secondary antibody at 1:200 for 1hr at room temperature. Cells were washed three times with PBS and mounted onto glass slides with Prolong gold anti-fade reagent with DAPI (Life Technologies). Staining was assessed using a Nikon ECLIPSE E600 immunofluorescent microscope and Velocity software. Cells with > 10 foci per cell were classed as foci positive. A minimum of 300 cells were counted for each experimental repeat. Representative images were taken with x100 magnification. Antibodies used include: γH2AX (mAB, Millipore), 53BP1 (Rabbit, Novus), RAD51 (Calbiochem), RPA (Calbiochem).

#### Cell Cycle Analysis and HR assay

HeLa cells were rinsed three times with phosphate buffered saline (PBS), and all media and wash buffer was retained to salvage mitotic cells. Cells were fixed dropwise in 70% ethanol (−20°C) while being gently vortexed, and incubated for at least one hour in 70% ethanol. DNA was stained with 25 μg/ml propidium iodide containing 0.1 mg/ml RNase A. Cells were analyzed using an Accuri flow cytometer (BD Biosciences) in conjunction with CFlowplus software. For analysis of G2/M checkpoint via phospho-Histone H3 (Ser10) (marker of mitotic cells), all cells (including mitotic cells) were harvested at specified time points and fixed in 70% ethanol prior to permeabilization with 0.25% Triton X-100 in 1% BSA/PBS. Cells were washed and incubated with pH3-Ser10 antibody (CST) for one hour at room temperature, followed by goat anti-rabbit-488 nm (Dylight) for 30 min. Cells were subsequently incubated in 25 μg/ml propidium iodide/0.1mg/ml RnaseA solution for 30 min and analyzed by flow cytometry. HR assays were performed as described previously (Butler et al., 2012).

#### TAP-Tag affinity purification

Catalytically inactive PRMT5 (G367A/R368A; PRMT5 MD) (Pal et al., 2004) was cloned into pcDNA 4/TO-C-TAP, and clonal tetracycline-regulated 293T/TR-C-tap PRMT5 MD cell lines generated. After 24 hr tetracycline treatment (1 μg/ml), cells were lysed in LB (150mM NaCl, 50mM Tris-HCl pH 8.0, 1mM EDTA, 1% NP40, 1mM Na_3_VO_4_, 50mM NaF, 1mM β-glycero-phosphate, 100 μM phenylmethylsulfonyl fluoride (PMSF), 10 μg/ml leupeptin, 10 μg/ml aprotinin), sonicated three times, and freeze/thawed twice. After clarification, lysates were incubated with IgG Sepharose 6 fast flow beads (GE Healthcare) for 4 hr at 4°C, washed three times in LB minus protease and phosphatase inhibitors, and once with TEV cleavage buffer (150mM NaCl, 10mM Tris pH 8.0, 0.5mM EDTA, 0.1% NP40, 1mM DTT). Complexes isolated by IgG Sepharose pull-down were released by two rounds of TEV protease cleavage (50 units; Promega) equating to a total time of 24 hr cleavage. IgG Sepharose beads were washed three times in CaBIND buffer (150mM NaCl, 50mM Tris pH 8.0, 2mM CaCl_2_, 1mM MgOAc, 1mM Imidazole, 0.1% NP40, 10mM β-mercaptoethanol) to ensure effective release of complexes, and elutions subjected to a second round of affinity purification using Calmodulin resin (Agilent). After three washes with CaBIND buffer, protein complexes were eluted with two rounds of EDTA buffer (150mM NaCl, 50mM Tris pH 8.0, 5mM EDTA, 0.1% NP40) at 4°C for 30 min. Proteins were precipitated with TCA, complexes resolved by SDS-PAGE, fixed and stained with SYPRO Ruby (Lonza).

#### Immunoprecipitation

For preparation of lysates for immunoprecipitation (IP), cells were washed three times in ice cold phosphate-buffered saline (PBS) and lysed in 0.1% NP40 Lysis buffer (150mM NaCl, 20mM Tris pH 7.5, 0.5mM Ethylenediaminetetraacetic acid (EDTA), 1mM Na_3_VO_4_, 50mM NaF, 1mM β glycero-phosphate, 100 μM phenylmethylsulfonyl fluoride (PMSF), 10 μg/ml Leupeptin, 10 μg/ml Aprotinin). Cells were sonicated twice at 25% amplitude for 5 s with a thin probe, and lysates cleared by centrifugation. Where appropriate, antibodies were added at a concentration of 1 μg/mg of lysate and incubated overnight at 4°C, followed by antibody-protein complex capture with Protein G Sepharose beads (GE Healthcare) for at least one hour at 4°C. Alternatively, lysates were directly incubated with anti-Flag M2-affinity beads (Sigma Aldrich). After extensive washing in NP40 lysis buffer, complexes were eluted and analyzed by SDS-PAGE and immunoblotting.

#### Mass Spectrometry

Polyacrylamide gel slices (1-2mm) containing the purified proteins were prepared for mass spectrometry analysis using the Janus liquid handling system (PerkinElmer). Briefly, the excised protein gel pieces were placed in a well of a 96-well microtiter plate and destained with 50% v/v acetonitrile and 50 mM ammonium bicarbonate, reduced with 10 mM DTT, and alkylated with 55 mM iodoacetamide. After alkylation, proteins were digested with 6 ng/μL endoproteinase Asp-N (Promega) overnight at 37°C. The resulting peptides were extracted in 2% v/v formic acid, 2% v/v acetonitrile. The digest was analyzed by nano-scale capillary LC-MS/MS using an Ultimate U3000 HPLC (ThermoScientific Dionex) to deliver a flow of approximately 300 nL/min. A C18 Acclaim PepMap100 5 μm, 100 μm x 20 mm nanoViper (ThermoScientific Dionex), trapped the peptides prior to separation on a C18 Acclaim PepMap100 3 μm, 75 μm x 250 mm nanoViper (ThermoScientific Dionex). Peptides were eluted with a 60 min gradient of acetonitrile (2% to 80%). The analytical column outlet was directly interfaced via a nano-flow electrospray ionization source, with a hybrid quadrupole orbitrap mass spectrometer (Q-Exactive Plus Orbitrap, ThermoScientific). Data dependent analysis was carried out using a resolution of 30,000 for the full MS spectrum, followed by ten MS/MS spectra. MS spectra were collected over a m/z range of 300–2000. MS/MS scans were collected using a threshold energy of 27 for higher energy collisional dissociation (HCD). LC-MS/MS data were then queried against a protein database (UniProt KB) using the Mascot search engine program (Matrix Science) (Perkins et al., 1999). Database search parameters were set with a precursor tolerance of 10 ppm and a fragment ion mass tolerance of 0.8 Da. One missed enzyme cleavage was allowed and variable modifications for oxidized methionine, carbamidomethyl cysteine, pyroglutamic acid, phosphorylated serine, threonine, and tyrosine, and methyl arginine were included. MS/MS data were validated using the Scaffold program (Proteome Software). All data were additionally interrogated manually.

#### Immunoblotting

Cells were treated with the pan methyltransferase inhibitor Adox (100 μM, Sigma) or the PRMT5 specific inhibitor GSK591 (5 μM, SGC, Oxford) (Chan-Penebre et al., 2015) for 24 hr prior to lysis. Whole cell extracts were obtained by lysis in UTB buffer (8 M Urea, 50 mM Tris, 150 mM β-mercaptoethanol), or in 0.1% NP40 lysis buffer (150mM NaCl, 20mM Tris pH 7.5, 0.5mM EDTA, 1mM Na_3_VO_4_, 50mM NaF, 1mM β-glycero-phosphate, 100 μM phenylmethylsulfonyl fluoride (PMSF), 10 μg/ml leupeptin, 10 μg/ml aprotinin). Cell extracts were sonicated twice followed by centrifugation to clarify lysate. Protein concentration was determined by Bradford Assay according to manufacturer’s instructions (BioRad). Protein lysates were resolved by SDS-PAGE, transferred on to PVDF and incubated with primary antibody overnight, followed by HRP-linked secondary antibody for 1hr at room temperature. The signal was detected using ECL western blotting substrate (Pierce).

#### In vitro PRMT1 methylation assay

All GST and His-tagged proteins were generated in-house using standards protocols. Histone H4 obtained from Cell Signaling Technologies. Purified recombinant GST–PRMT1 was incubated with 2 μg substrate and 1 μL S-[methyl-^3^H]–adenosyl-L-methionine (^3^[H]-SAM) (specific activity: 55–85Ci/mmol, PerkinElmer) in a total volume of 60 μL supplemented with 100mM sodium phosphate (pH 7.5) buffer. Reactions were incubated at 37°C for 1 hr and denatured protein resolved by SDS–PAGE. Protein was transferred onto nitrocellulose membrane, and the tritium signal enhanced by treating membranes with EN3HANCE (PerkinElmer). Membranes were exposed to autoradiography film for at least 1 month at −80°C.

#### In vivo methylation assays

Transfected cells were cultured in methionine-free DMEM (Sigma Aldrich) supplemented with 10% fetal calf serum (FCS) and 1% glutamine. To inhibit de novo protein synthesis, cycloheximide (100 μg/ml) and chloramphenicol (40 μg/ml) were added for one hour prior to labeling with L-[Methyl-^3^H]-methionine (specific activity 70-85Ci (2.59-3.145 TBq)/mmol, 10 μCi/ml media; Perkin Elmer) for four hours. Cells were harvested and lysed in RIPA buffer (50mM Tris pH 7.4, 150mM NaCl, 1mM EDTA, 1% NP40, 0.5% sodium deoxycholate 0.1% SDS, 10% glycerol, 1mM PMSF, 50mM NaF, 10mM Na_3_VO_4_, 1 μg/ml leupeptin and 1 μg/ml aprotinin), and Flag proteins immunoprecipitated overnight using anti-Flag M2 affinity beads (Sigma). After four washes with RIPA buffer, immunoprecipitates were denatured, resolved by SDS-PAGE and transferred onto nitrocellulose membrane. To verify equal immunoprecipitation, one-tenth of the immunoprecipitation was retained for western blot analysis. To enhance tritium signal, membranes were treated with EN3HANCE (Perkin Elmer), and exposed to autoradiography film for 2-4 weeks at −80°C.

#### In vitro HAT assay

TIP60 HAT assays were conducted as described (Jacquet et al., 2016). Briefly, 293T cells were transfected with the TIP60 piccolo complex (plasmids were a kind gift from Bruno Amati, European Institute of Oncology, Milan) and Flag-TIP60 immunoprecipitated from cell lysates. HAT assay was conducted at 37°C for 1 hr with 1 μg recombinant Histone H4 and 150 μM acetyl CoA in assay buffer (50mM Tris pH 8.0, 10% glycerol, 1mM EDTA, 1mM DTT, 1mM PMSF, 10mM sodium butyrate). Acetylated H4 was detected by immunoblotting with penta-H4-Ac (Abcam).

#### Quantitative PCR

RNA was extracted from cells using the QIAGEN RNeasy mini kit, DNase treated (Ambion) and cDNA synthesized using Superscript III (Invitrogen) according to the manufacturer’s instructions. QPCR was performed using a Stratagene Mx3005P detection system with SYBR Green incorporation with primers indicated below.

**Table:** 

Gene	Forward Primer	Reverse Primer
TIP60	5′-CAATGTGGCCTGCATCCTA-3′	5′-ATGAACTCTCCAAAGTGGAAGG-3′
MOF	5′-AAGTCACGGTGGAGATCGG	5′- GCTGAAGTGATCCAGTCTCG-3′
actin	5′-CTCTTCCAGCCTTCCTTCCT-3′	5′- GAAGTGTGACGTGGACATCC −3′
INO80	5′-GCCCCCTTCCATGTGGTTAT-3′	5′-GGATCTTCCAACGAACACTGG-3′
BRCA2	5′-CCTGATGCCTGTACACCTCTT-3′	5′-GCAGGCCGAGTACTGTTAGC-3′
SRCAP	5′-CTACTCCAGGGCCCACTACT-3′	5′-AATGGGCGTCTTTACCTGGG-3′

All primer pairs spanned exon-intron boundaries and generated a single product as determined by dissociation curve analysis.

#### Chromatin Immunoprecipitation

Chromatin immunoprecipitation was performed as described (McNee et al., 2016). Briefly, U20S-265 cells treated for 4 hr with 4-OHT (10 μM, Sigma) and the Shield-1 ligand (0.5 μM, Promega) were crosslinked with 1% formaldehyde before neutralization with 0.125 M glycine. After lysis and sonication, chromatin was pre-cleared using normal rabbit IgG (Millipore), or mouse IgG (Cell Signaling Technology), and Protein G-Sepharose (Roche) before co-immunoprecipitation with either H4K16Ac (Millipore), 53BP1 (Novus) or RUVBL1 (Sigma SAB4200184). After washing and elution of protein-DNA complexes from the beads (100 mM NaHCO_3_, 1% SDS), crosslinks were reversed by heating, and treated with proteinase K. Associated DNA was quantified by qPCR analysis using the primers listed below (Tang et al., 2013).

**Table:** 

p1	5′-GGAAGATGTCCCTTGTATCACCAT-3′	5′-TGGTTGTCAACAGAGTAGAAAGTGAA-3′
p2	5′- GCTGGTGTGGCCAATGC-3	5′-TGGCAGAGGGAAAAAGATCTCA-3′
p3	5′-GGCATTTCAGTCAGTTGCT CAA-3′	5′- TTGGCCGATTCATTAATGCA
p4	5′-CCACCTGACGTCTAAGAAACCAT-3′	5′-GATCCCTCGAGGACGAAAGG-3′

All primer pairs generated a single product as determined by dissociation curve analysis.

### Quantification and Statistical Analysis

All statistical analysis was carried out using Student’s t test. Unless stated, ^∗^p < 0.05; ^∗∗^p < 0.005; ^∗∗∗^ p < 0.0005; ^∗∗∗∗^ p < 0.00005, as stated within the figure. n = number of experimental replicates.

## Author Contributions

T.L.C. and C.C.D. performed the experiments and designed the research. M.P.S.-B., K.C., and J.H.-R. contributed to immunoblot analysis, fluorescence-activated cell sorting (FACS) analysis, and ChIP. J.J.R. performed chromatid break analysis. S.L.M. and J.M.S. conducted and analyzed mass spectrometry data. T.M.B. and P.M.M. provided structural advice and in silico modeling of RUVBL1-R205me2s. J.J.R. and G.S.S. advised on experiments. C.C.D. and G.S.S. wrote the manuscript.

## Supplemental Information

Document S1. Figures S1–S7

Document S2. Article plus Supplemental Information
