# Supplementary material for: PRMT5-Dependent Methylation of the TIP60 Coactivator RUVBL1 Is a Key Regulator of Homologous Recombination
Source: Mol Cell. 2017 Mar 2;65(5):900–916.e7. doi: 10.1016/j.molcel.2017.01.019 (PMC5344794; doi:10.1016/j.molcel.2017.01.019)
Supplement: Document S1. Figures S1–S7 [file mmc1.pdf]

Molecular Cell, Volume *65*

Supplemental Information

PRMT5-Dependent Methylation of the TIP60 Coactivator RUVBL1 Is a Key Regulator of Homologous Recombination

Thomas L. Clarke, Maria Pilar Sanchez-Bailon, Kelly Chiang, John J. Reynolds, Joaquin Herrero-Ruiz, Tiago M. Bandeiras, Pedro M. Matias, Sarah L. Maslen, J. Mark Skehel, Grant S. Stewart, and Clare C. Davies

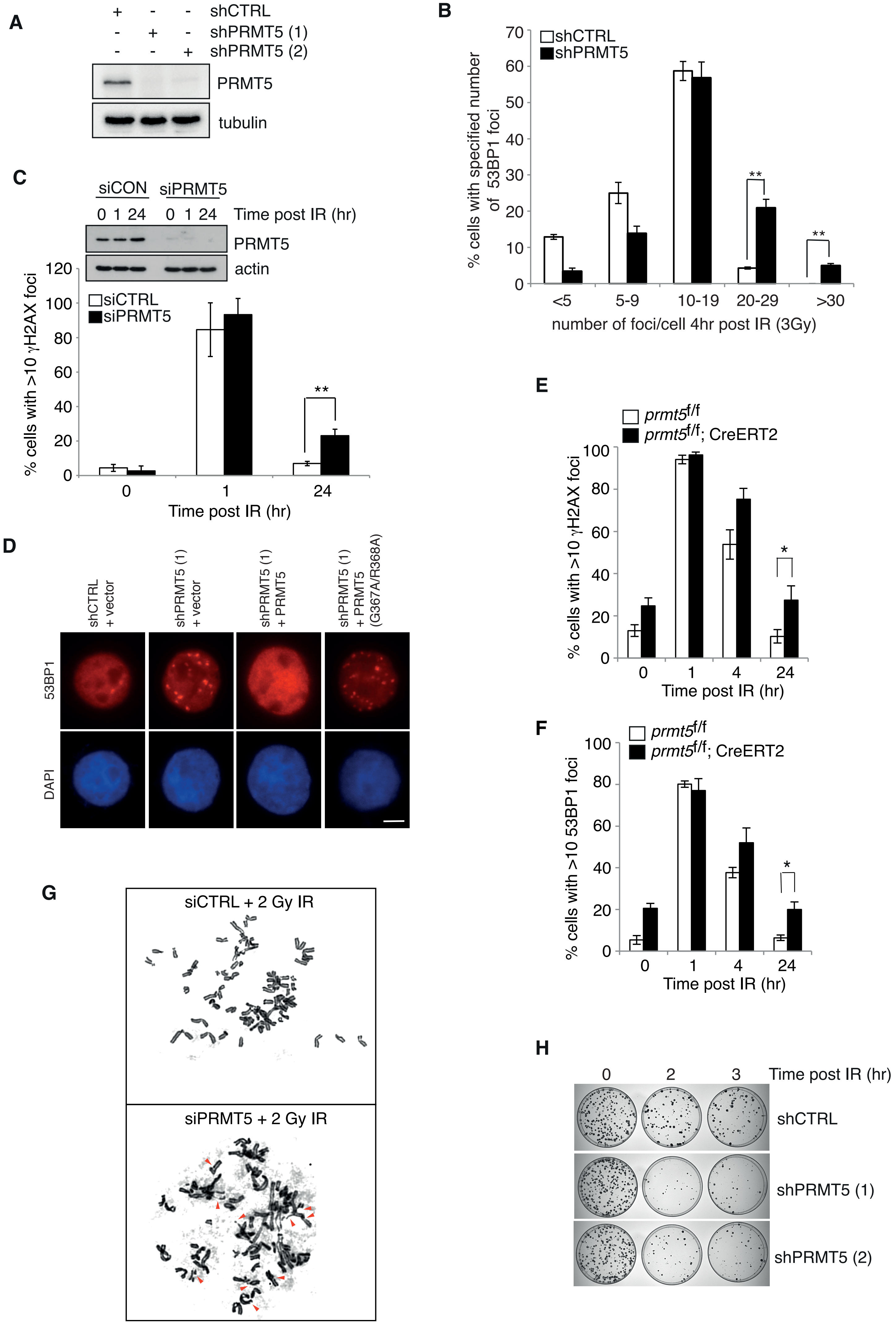

Clarke et al: Supplementary Figure 1

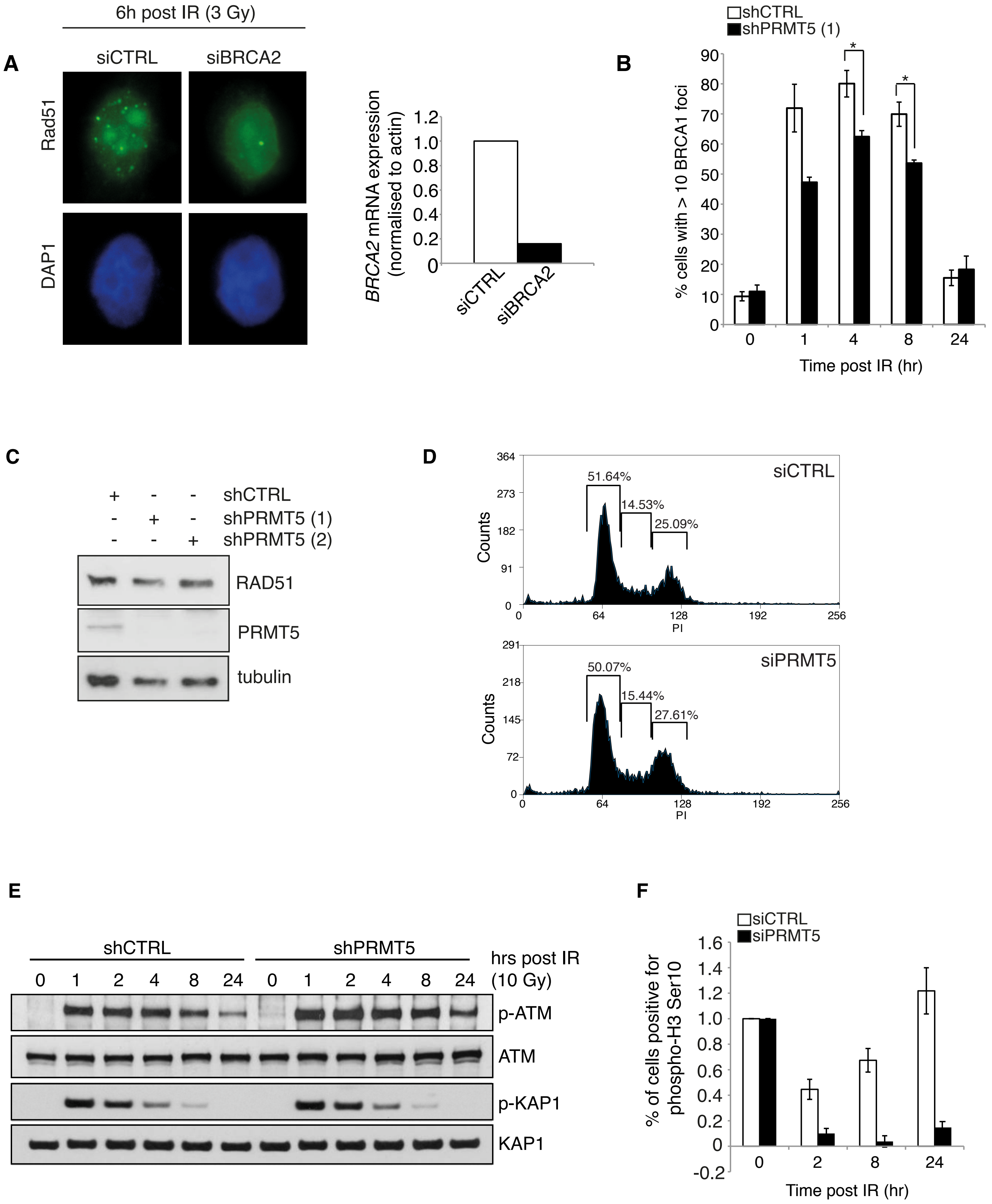

Clarke et al: Supplementary Figure 2

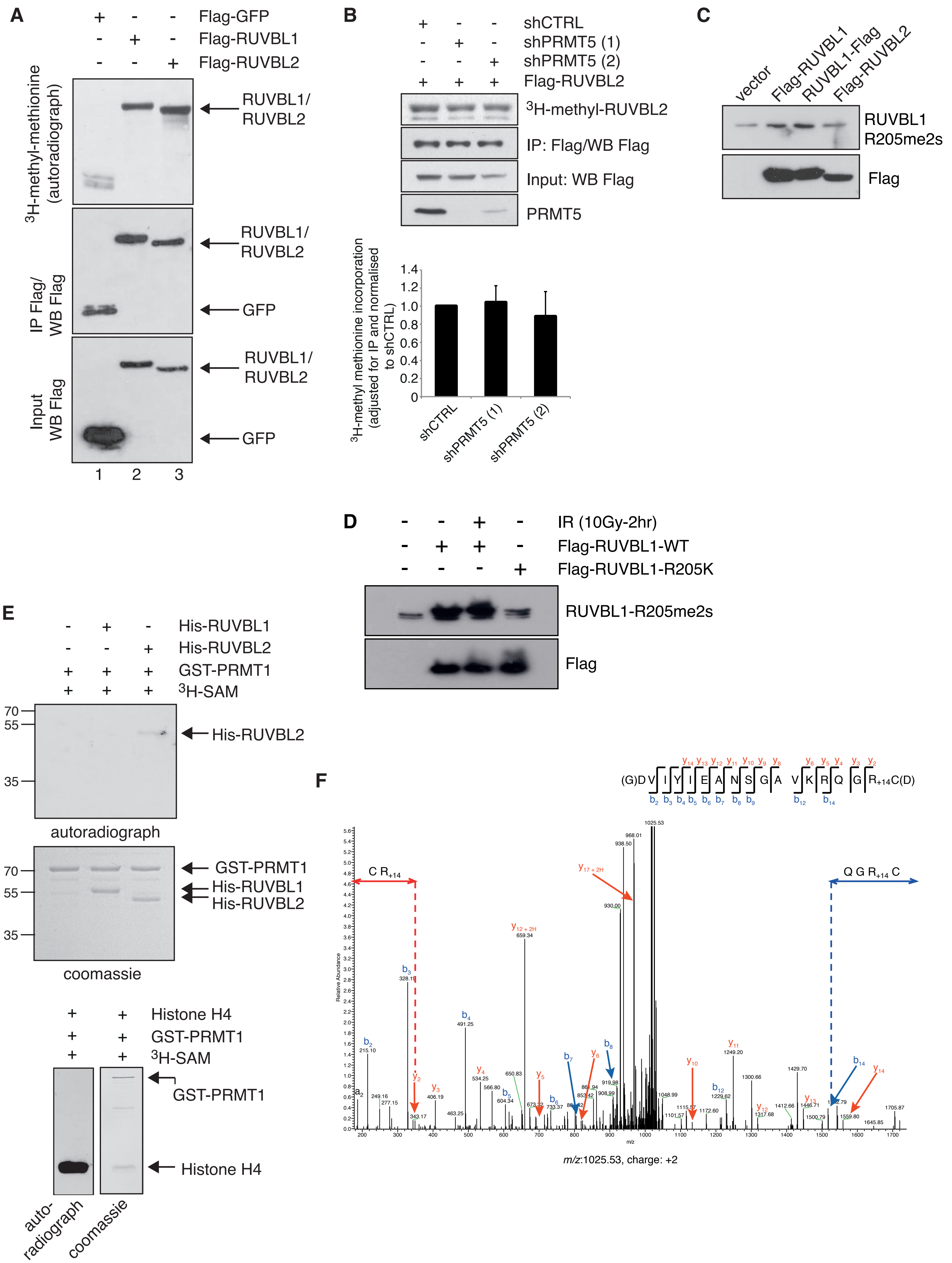

Clarke et al: Supplementary Figure 3

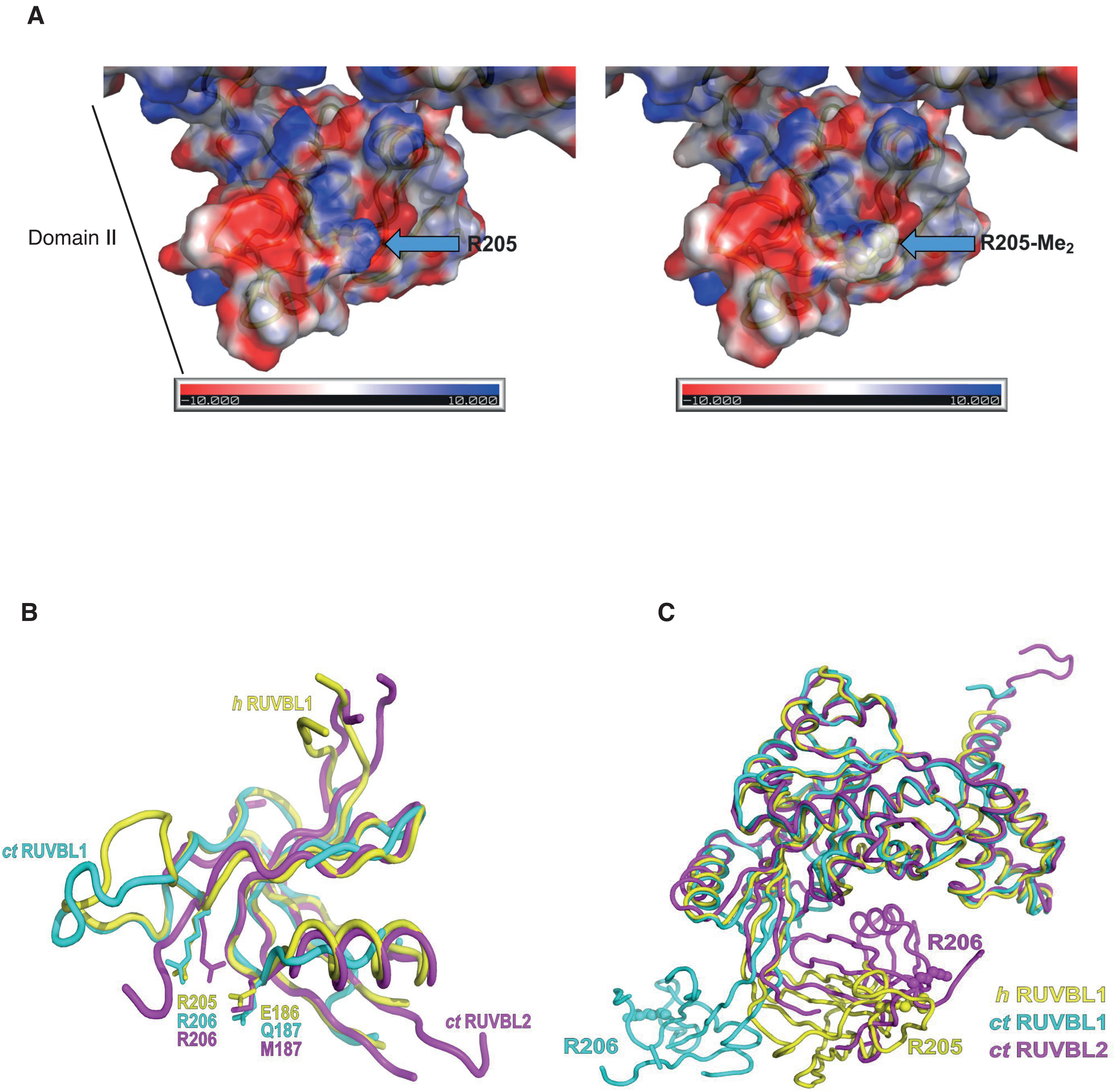

Clarke et al: Supplementary Figure 4

A

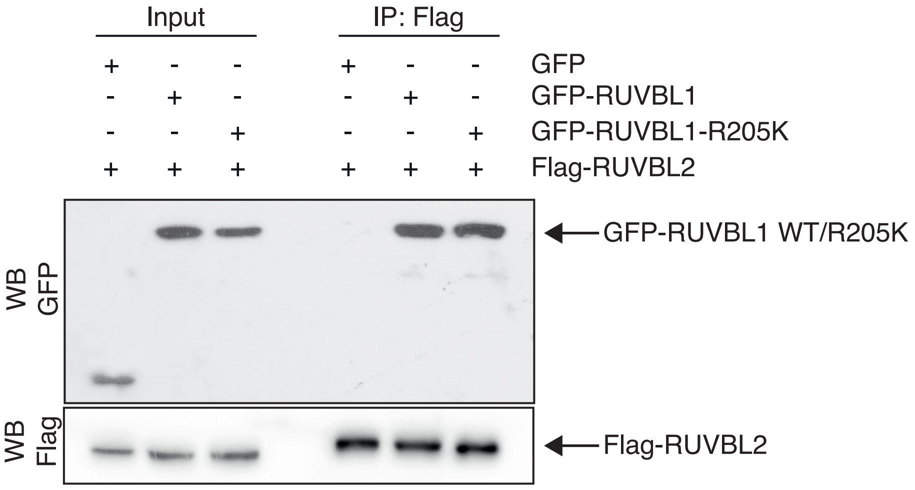

B

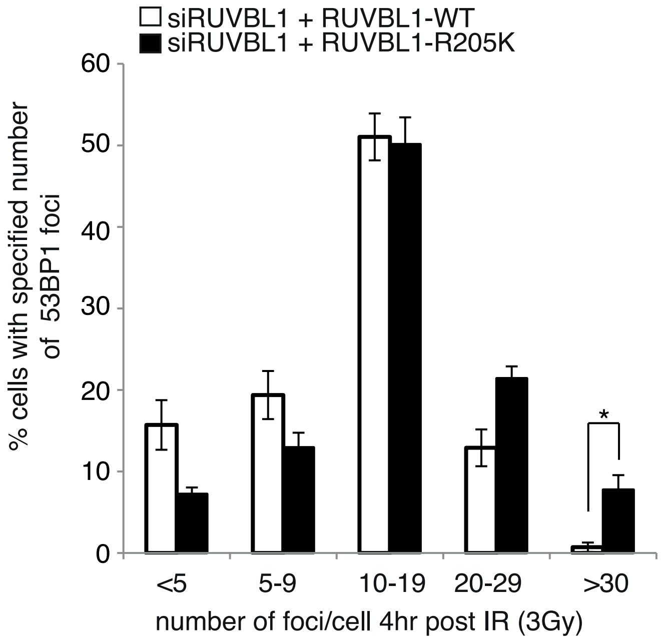

C

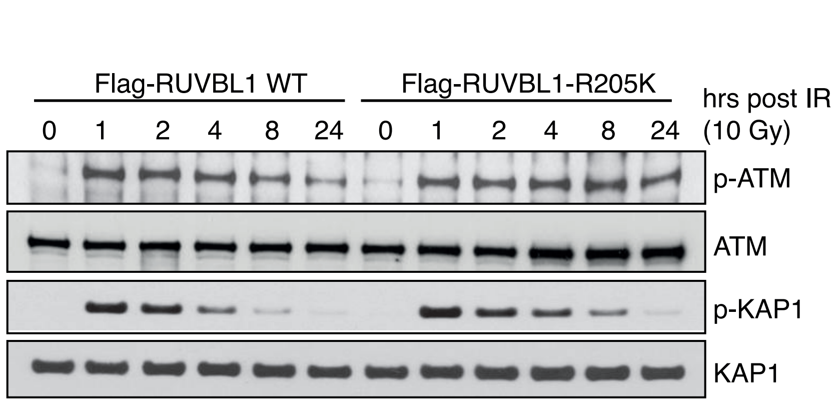

Clarke et al: Supplementary Figure 5

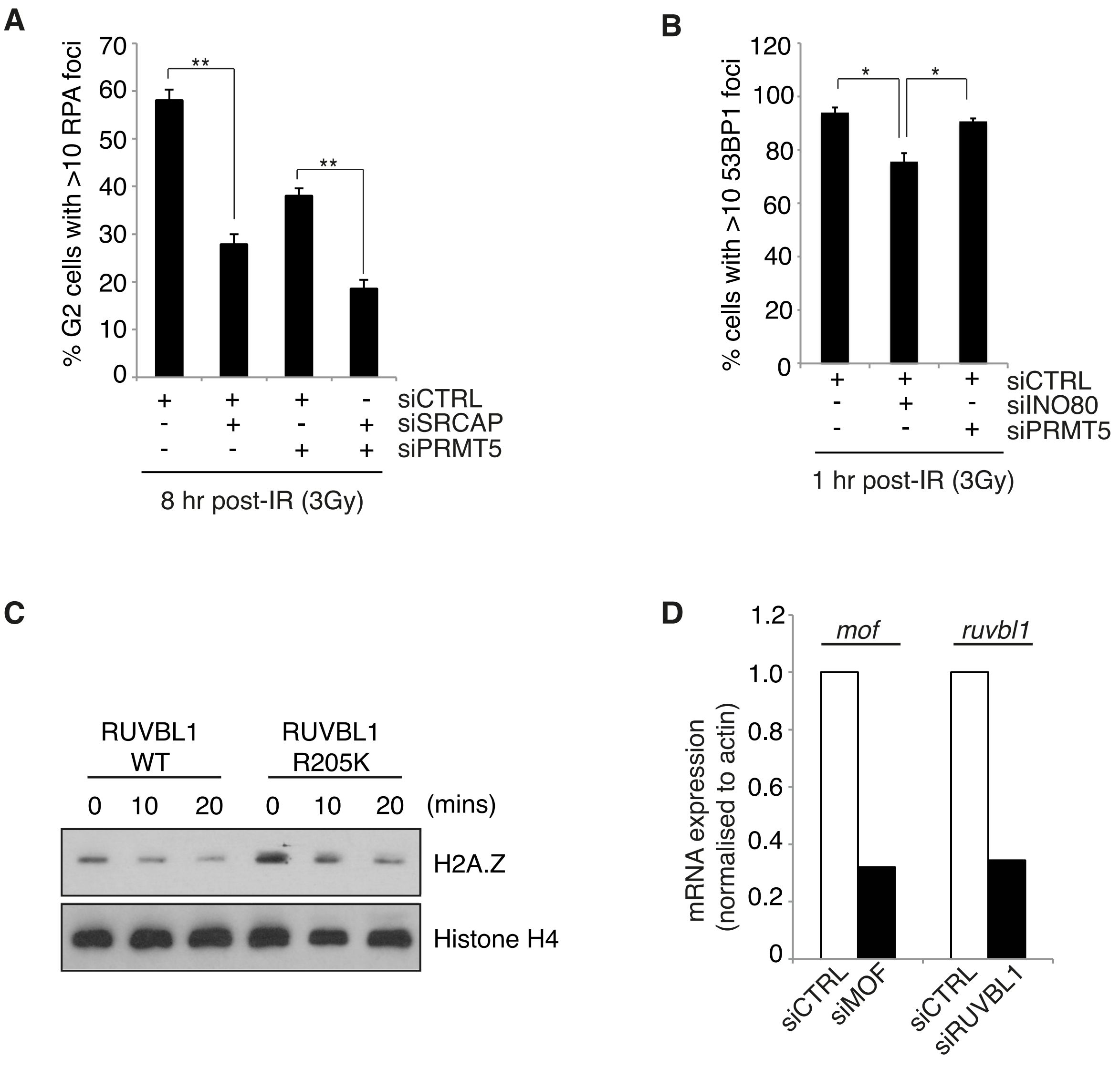

Clarke et al: Supplementary Figure 6

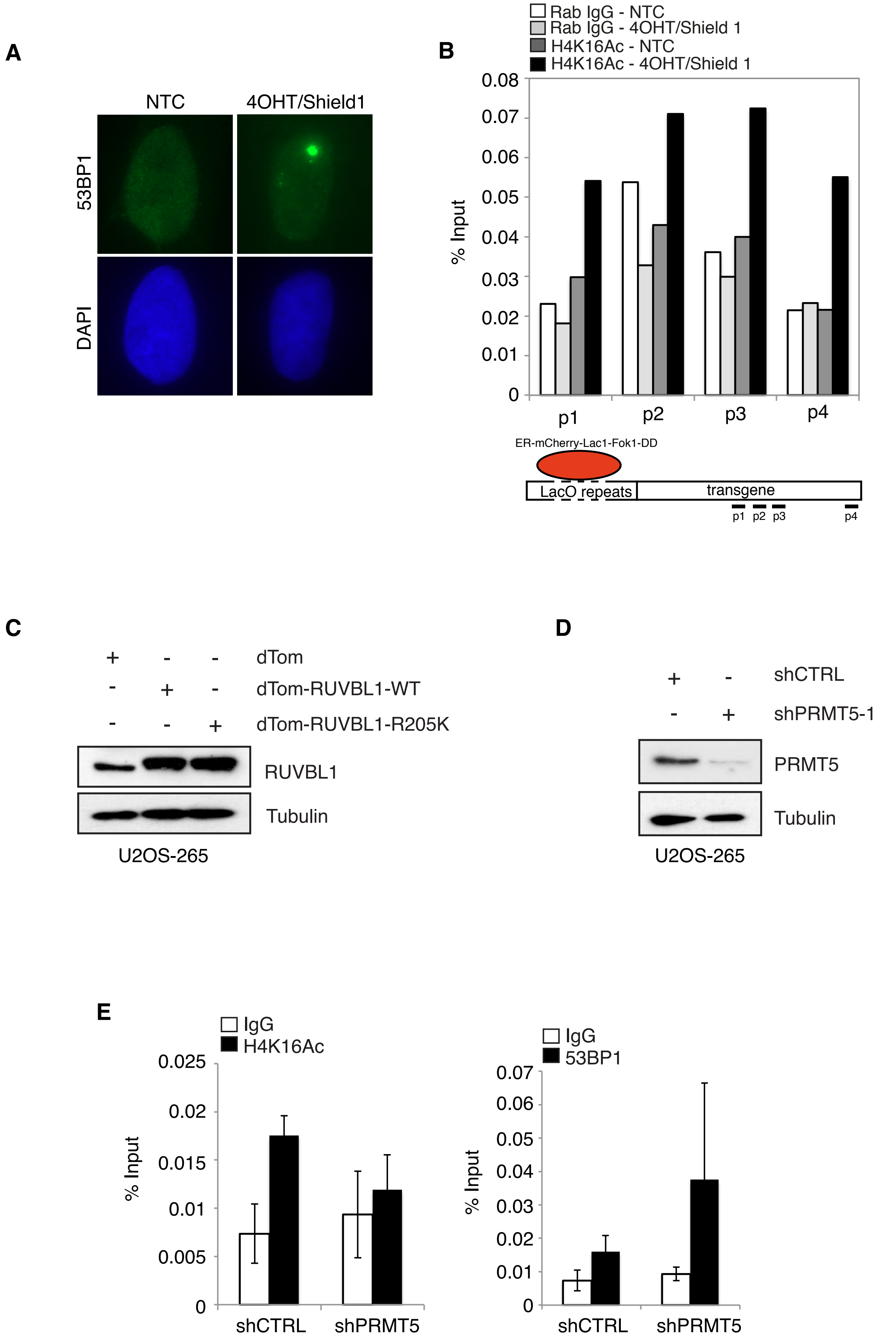

Clarke et al: Supplementary Figure 7

Supplementary Figure S1, related to Figure 1. The effects of PRMT5 depletion on DSB repair

(**A**) Generation of stable PRMT5 depleted HeLa cells. shPRMT5 (1) and shPRMT5 (2) represent two independent polyclonal cell lines that express distinct shRNA sequences directed towards PRMT5. **(B)** Stratification of the number of 53BP1 foci 4hr post IR (3Gy) represented in Figure 1C. (**C**) Upper panel: validation of transient siPRMT5 knockdown by immunoblot analysis. Lower graph: HeLa cells were transfected with siRNA, and exposed to 3Gy IR 48hrs later. Cells were taken at the time points indicated and γH2AX foci formation determined by immunofluorescence staining. Cells with >10 foci were scored (mean ± s.e.m. n = 3). (**D**) Representative images of 53BP1 foci formation as quantified in Figure 1D. (**E**) and **(F)** Primary, non-immortalised *prmt5*$^{f/f}$ and *prmt5*$^{f/f}$;CreERT2 mouse embryonic fibroblast (MEFs) were cultured in the absence or presence of 4-hydroxy-tamoxifen (4-OHT; 500nM for 24hrs), followed by 4 days in 4-OHT deficient media. Cells were damaged with 5Gy IR and the number of γH2AX **(E)** and 53BP1 foci **(F)** quantified (mean ± s.e.m. * P <0.05, n=3). (**G**) Representative image of chromosomal damage after IR-induced damage and PRMT5 depletion. Quantification of image is in Figure 1J. (**H**) Representative image of colony formation as quantified in Figure 1I.

Supplementary Figure S2, related to Figure 2. PRMT5 regulates HR-mediated DSB repair

(A) Validation of RAD51 antibody. Depletion of BRCA2 reduces the number of foci as identified by the RAD51 antibody used in this study. Right panel: validation of BRCA2 knockdown. **(B)** Analysis of BRCA1 foci in Hela-shCTRL and HeLa-shPRMT5 (1) cells after 3Gy IR. Cells with >10 foci were scored (mean ± s.e.m. n = 3). **(C)** Immunoblot analysis of Rad51 expression in stable HeLa cell lines. **(D)** Cell cycle profiles of undamaged HeLa-shCTRL and shPRMT5 cells. **(E)** ATM signaling is unchanged in PRMT5 depleted cells. **(F)** Analysis of G2/M checkpoint via the mitotic marker phospho-Histone H3 (Ser10).

Supplementary Figure S3, related to Figure 3. Identification of RUVBL1 as an arginine methylated protein

(A) 293T cells were transfected with Flag-RUVBL1, Flag-RUVBL2 or Flag-GFP (negative control), treated with cycloheximide (CHX) and chloramphenicol (CAM), and labeled with [^{3}H]-methyl methionine. Flag-tagged proteins were immunoprecipitated and incorporated methyl groups detected by SDS-PAGE followed by autoradiograph. (**B**) Transfected Hela-shCTRL or shPRMT5 cell lines were treated with CHX/CAM and labeled with [^{3}H]-methyl methionine. Flag-RUVBL2 was immunoprecipitated and incorporated methyl groups detected by SDS-PAGE followed by

autoradiograph. Quantification of [^{3}H]-methyl signal intensity adjusted for IP and normalised to shCTRL (mean ± s.d). (**C**) and (**D**) Transfected 293T cells were lysed and immunoblotted with the RUVBL1 methyl-specific antibody, RUVBL1-R205me2s. (**E**) *In vitro* PRMT1 methylation assay demonstrating that histone H4 (bottom panel) but not RUVBL1 (top panel) is effectively methylated by PRMT1. Recombinant GST-tagged PRMT1, His-tagged RUVBL1, His-tagged RUVBL2 or untagged histone H4 were incubated with [^{3}H]-SAM and proteins separated by SDS-PAGE. Incorporation of radiolabelled methyl groups was detected by autoradiograph. Coomassie stain indicates protein levels. (**F**) Higher energy Collisional Dissociation (HCD) MS/MS spectrum and associated peptide sequence of the arginine methylated peptide DVIYIEANSGAVKRQGRC, residues 203-220 m/z 1025.53 $^{2+}$, derived from RUVBL1. The identified b and y ions are annotated on the spectrum. The y2 ion shows a +14-amu shift suggesting methylation.

Supplementary Figure S4, related to Figure 3. Structural orientation of RUVBL1-R205

(**A**) Effect of methylation on the electrostatic potential of homohexameric RUVBL1 mapped at the molecular surface. Wild type RUVBL1 (left) and RUVBL1 with methylated R205 (right) modelled in the same conformation as in wild type RUVBL1. Calculations with PDB2PQR (Dolinsky et al., 2004) and APBS (Baker et al., 2001). Charges for methylated R205 were adapted from Mulliken charges calculated using a B3LYP/6-31G(d) basis set with geometry optimisation. The EP surface is semitransparent and coloured from blue to red to represent values between ‑10 and 10 kT/e. The C (α) trace of a RUVB1 monomer is shown in tube representation and R205 is shown as spheres, including hydrogen atoms in calculated positions. (**B**) Location of R205 in superimposed Domains II of homohexameric human RUVBL1 (PDB 2c9o; yellow chain) and hetero-hexameric RUVBL1/RUVBL2 complex from *chaetomium thermophilum* (PDB 4ww4; cyan and magenta chains, respectively). Residues E186 in human RUVBL1, Q187 in ct RUVBL1 and M187 in ct RUVBL2 are also represented as sticks. Crystal structures taken from Matias et al. (2006) and Lakomek et al. (2015). (**C**) The flexible orientation of the DII extension in RUVBL1 and RUVBL2. Superimposed structures of RUVBL1 and RUVBL2 monomers in homohexameric human RUVBL1 (PDB 2c9o; yellow chain) and hetero-hexameric RUVBL1/RUVBL2 complex from *chaetomium thermophilum* (PDB 4ww4; cyan and magenta chains, respectively).

Supplementary Figure S5, related to Figure 3, 4 and 5. Mutation of R205 to lysine does not interfere with RUVBL1/RUVBL2 interactions or ATM activation, but does promote retention of 53BP1 at break ends 4hrs after IR-induced DSBs

(A) 293T cells were transfected with Flag-tagged RUVBL2 and GFP-tagged wild type or R205K mutated RUVBL1, and RUVBL2 immunoprecipitated by anti-Flag antibodies. Associated RUVBL1 was detected by SDS-PAGE and immunoblotting using the GFP antibody. **(B)** Reconstitution of RUVBL1 depleted cells with methyl-deficient RUVBL1 (R205K) leads to an increase in the number of cells displaying high levels of 53BP1 foci 4hr after IR-induced damage (3Gy). Foci number were stratified into 5 groups (<5, 5-9, 10-19, 20-29 and >30 foci per cell) (mean ± s.e.m. * P <0.05, n=3). **(C)** ATM signaling is unchanged in RUVBL1-R205K reconstituted cells.

Supplementary Figure S6, related to Figure 6 and Figure 7. PRMT5 is not epistatic with INO80 or SRCAP during IR-induced DSB repair

(A) HeLa cells were transfected with either siPRMT5, siSRCAP, or both, and damaged with 3Gy IR. 8 hrs post-IR, the number of cells with >10 RPA foci were quantified (mean ± s.e.m. n=3). **(B)** HeLa cells were transfected with either siPRMT5 or siINO80, and damaged with 3Gy IR. 1 hr post-IR, the number of cells with >10 53BP1 foci were quantified (mean ± s.e.m. n=3). **(C)** HeLa-RUVBL1 WT or R205K expressing cells were damaged with 10Gy IR and chromatin isolated. H2A.Z loading was determined by immunoblot analysis. (**D**) Validation of TIP60 and MOF knockdown. HeLa-shCTRL cells were transfected with siMOF or siRUVBL1, and RNA extracted 48hrs later. *mof* and *ruvbl1* transcript levels were determined by quantitative PCR and normalised to *actin* mRNA expression.

Supplementary Figure S7, related to Figure 7. Validation of ER-mCherry-Lac1-Fok1-DD induction of double strand breaks and generation of cells lines used in this study

(A) U2OS-265 cells stably express a DSB reporter system that uses an mCherry-Lac1-Fok1 nuclease fusion protein to create DSBs within a defined single genomic locus (Shanbhag et al., 2010; Tang et al., 2013)**.** Treatment of cells with 4-OHT (10μM) and the Shield-1 ligand (0.5μM) enables controlled nuclear expression of the fusion protein and the controlled induction of DSB that can be visualised as a large single 53BP1 focus. **(B)** Fok1-induced DSB promotes H4K16 acetylation, as determined by chromatin immunoprecipitation (ChIP). p1-p4 represent primer pairs spanning the transgene downstream of the LacO repeats targeted by Fok1. **(C)** Generation of U2OS-265-dTom-RUVBL1 wild type (WT) and R205K expressing cells. **(D)** Generation of U2OS-265 shCTRL and shPRMT5 expressing cells. (**E**) ChIP performed with H4K16Ac and 53BP1 antibodies in the presence of mCherry-Lac1-Fok1-DD-induced DSBs, in U2OS-265-shCTRL or shPRMT5 expressing cells. Data represents average % input across the four primer pairs (mean ± s.e.m. n = 3).
